# A Light‐Driven Closed‐Loop Chemical Recycling System for Polypinacols

**DOI:** 10.1002/adma.202506733

**Published:** 2025-06-29

**Authors:** Ahsen Sare Yalin, Patrick Schara, Željko Tomović, Fabian Eisenreich

**Affiliations:** ^1^ Department of Chemical Engineering and Chemistry Institute for Complex Molecular Systems Eindhoven University of Technology Eindhoven MB 5600 The Netherlands

**Keywords:** chemical recycling, photocatalysis, photochemistry, pinacol coupling

## Abstract

The development of innovative recycling strategies for polymers is crucial to addressing the rapidly growing plastic waste challenge. While thermal ground‐state chemistry is the standard for closed‐loop chemical recycling, the potential of photochemical excited‐state chemistry remains largely unexplored. This study bridges this gap by investigating light‐driven polymerization and depolymerization processes for hydroxyl‐rich polymers. Through consecutive pinacol coupling reactions, a range of simple bis‐aldehyde monomers is photopolymerized into well‐defined polypinacols on a gram scale. These polymers exhibit excellent thermal stability, retaining their integrity up to 306 °C, with glass transition temperatures ranging from 72 to 137 °C. Using an earth‐abundant cerium photocatalyst, selective cleavage of stable C─C bonds within the polypinacol backbone is achieved under visible light, efficiently regenerating the original monomer. As this approach tolerates the presence of standard commodity plastics, it presents an opportunity for orthogonal recycling methods that could help recover specific polymers from diverse plastic waste streams. The successful completion of one recycling cycle, resulting in a polymer with comparable properties to the original, highlights the significant potential and advantages of (photo)chemical recycling.

## Graphical abstract

This study presents a novel strategy for the photochemical closed‐loop recycling of hydroxyl‐rich polymers. Simple bis‐aldehyde precursors are employed to synthesize polypinacols via UV light irradiation. Subsequent depolymerization is achieved under visible light using a cerium‐based photocatalyst, enabling monomer recovery through selective cleavage of C─C bonds within the polymer backbone.

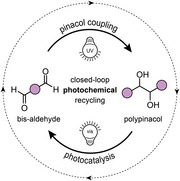

## 1 Introduction

With plastic pollution reaching unprecedented levels, developing sustainable recycling solutions has become a top global priority. Yet, the majority of plastic waste ends up in landfills, is incinerated, or leaks into the environment, while only a small portion undergoes chemical recycling.^[^ ^1^, ^2^ ^]^ Chemical recycling enables the conversion of polymers into virgin monomers, representing the ideal scenario for a closed‐loop recycling system.^[^ ^1^, ^3^ ^]^ Unlike mechanical recycling, which leads to a gradual loss of material properties,^[^ ^4^ ^]^ and pyrolysis, which suffers from low selectivity and high energy demand,^[^ ^5^ ^]^ this approach offers a more precise and effective solution.

As the need for sustainable recycling solutions grows, light‐driven strategies are gaining momentum as a transformative alternative within chemical recycling.^[^ ^6^ ^]^ Light, an abundant and renewable energy source, offers exceptional tunability across multiple dimensions—wavelength, intensity, and duration—enabling precise control over reaction pathways and selectivity.^[^ ^7^ ^]^ In contrast to traditional thermal methods, photochemical reactions typically proceed under ambient temperature and pressure, making them inherently more energy‐efficient and less prone to unwanted byproducts. Furthermore, the ability to excite molecules to higher energy states unlocks unique reactivity pathways that are fundamentally distinct from ground‐state thermal processes. In this context, photocatalysis stands out as a powerful technique, enabling the efficient cleavage of robust C─C bonds that are fundamental to the structure of most polymer backbones.^[^ ^8^, ^9^ ^]^

A growing number of seminal light‐driven depolymerization strategies has been reported in recent years that move beyond mere photodegradation to CO_2_.^[^ ^10^, ^11^, ^12^ ^]^ The Knowles group demonstrated the selective scission of C─C bonds in linear polymer chains, such as phenoxy resins and hydroxylated polyolefins, using an iridium photocatalyst through a proton‐coupled electron transfer under visible light irradiation.^[^ ^13^ ^]^ They later expanded this strategy to crosslinked thiol‐epoxy networks, employing an organic acridinium photocatalyst.^[^ ^14^ ^]^ Impressively, the depolymerization proceeded effectively despite the thermoset remaining insoluble in the reaction medium. These studies yielded either aromatic small molecules or aliphatic oxygenated compounds. Similarly, the Soo group developed a photocatalytic cascade depolymerization method for various hydroxy‐terminated polymers, such as polyethylene glycol, using a vanadium photocatalyst.^[^ ^15^ ^]^ This process generated chemical fuels and feedstocks, including formic acid and methyl formate. Beyond hydroxylated polymers, conventional polystyrene was also extensively investigated. A diverse array of photocatalytic systems, ranging from iron salts,^[^ ^16^, ^17^, ^18^, ^19^ ^]^ strong acids,^[^ ^20^ ^]^ to organic dyes,^[^ ^21^ ^]^ were developed to convert polystyrene effectively into benzoic acid under light illumination.

All of these photocatalytic depolymerization methods have in common that they generate organic molecules distinct from the original monomers used in polymer synthesis. Consequently, they are classified as upcycling strategies. However, a true closed‐loop photochemical recycling system, where polymers are depolymerized back into their original monomers and then repolymerized, driven entirely by light, has yet to be achieved. Realizing such a system requires a photochemical reaction that not only forms stable C─C bonds between simple organic molecules to construct the polymer backbone but also introduces functional groups that facilitate the selective cleavage of these covalent bonds, ultimately enabling the recovery of the original monomers.

We thus propose to utilize pinacol coupling reactions—one of the most extensively studied photochemical transformations—first discovered by Ciamician in 1900.^[^ ^22^ ^]^ Pinacol coupling reactions describe the reductive coupling of carbonyl groups via light illumination to form 1,2‐diols, also known as pinacol. Despite its long‐standing presence and well‐documented success in small molecule chemistry, the application of light‐driven pinacol coupling reactions for polymerizations has remained largely unexplored. In the 1970s, attempts were made to synthesize macromolecules, termed polypinacols, from carbonyl monomers, but these attempts showed very little success.^[^ ^23^, ^24^, ^25^ ^]^ The resulting polymers exhibited low molecular weights, comprising only five or fewer monomers. Additionally, these methods were restricted to ketones, with aldehydes being excluded, and the use of harsh, intense UV light led to polymer degradation.^[^ ^25^ ^]^ At that time, photochemical pinacol coupling reactions were not as extensively explored as they are today, with illumination systems primarily limited to inefficient mercury lamps. These constraints likely hindered deeper investigation into this promising field. However, the advent of operationally simple LED technology and a renewed interest in photochemistry have elevated pinacol coupling reactions to a powerful tool in synthetic chemistry. Recently, a wide range of light‐driven protocols has been developed to efficiently link small carbonyl molecules under mild conditions,^[^ ^26^, ^27^, ^28^, ^29^, ^30^ ^]^ while providing an exceptional level of control.^[^ ^31^, ^32^, ^33^ ^]^ On top of that, recent advancements in photocatalysis have shown that robust C─C bonds adjacent to hydroxyl groups can be cleaved selectively.^[^ ^34^ ^]^

**Figure 1:**
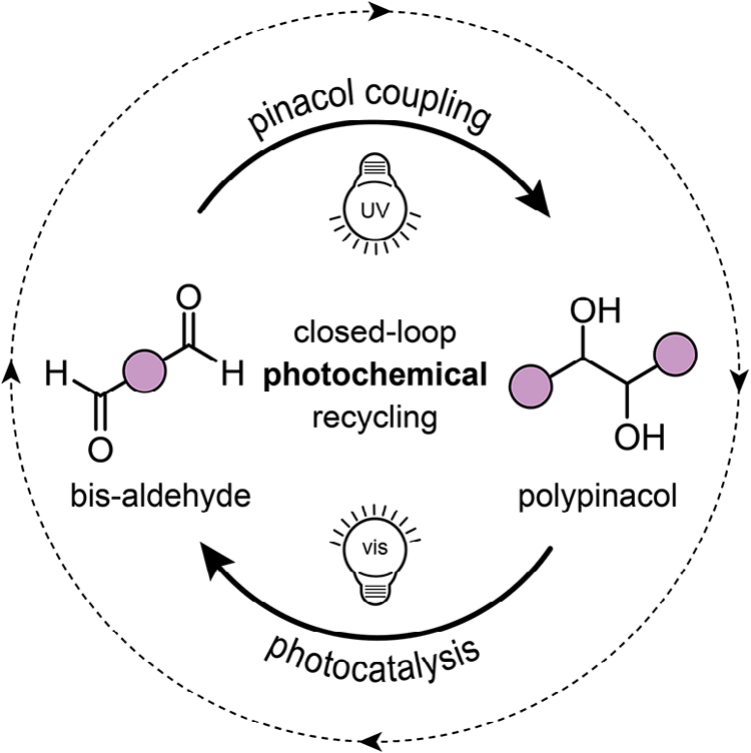
Illustration of a novel strategy for closed‐loop photochemical recycling of polypinacols.

In this work, we present the first fully light‐driven closed‐loop recycling system for polypinacols, where both polymerization and depolymerization are powered by light and successfully applied to linear polymers (**Figure** **1**). Consequently, this study closes a critical gap in photochemical recycling technologies and pushes the boundaries of sustainable polymer science.

## 2 Result and Discussion

### 2.1 Photopolymerization via Pinacol Coupling Reactions

**Figure 2:**
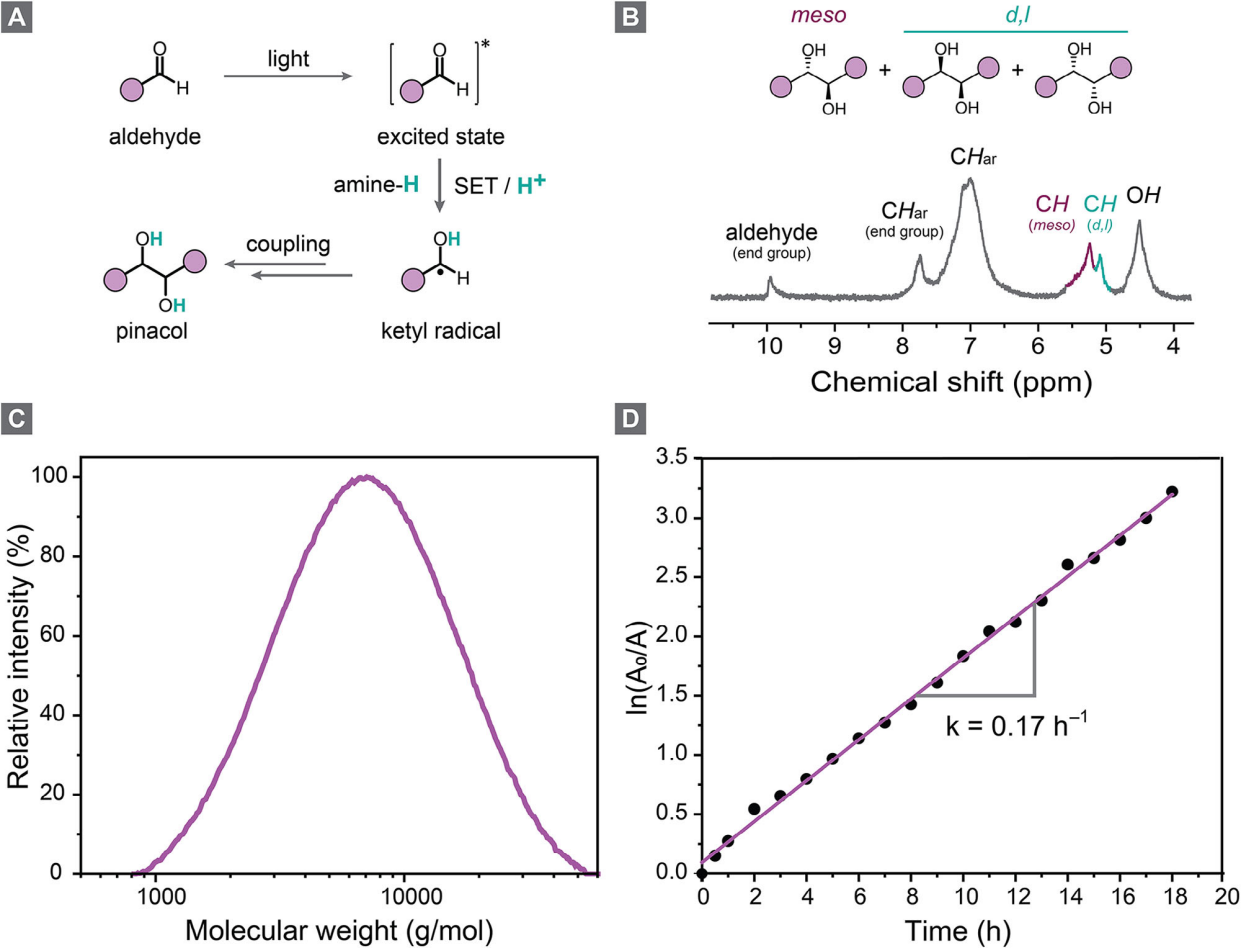
a) Simplified mechanism of the photochemical pinacol coupling, b) ^1^H‐NMR spectrum of polypinacol **P1** in DMSO‐d_6_. c) GPC measurement of **P1**. d) Kinetic profile of the polymerization of **M1**, determined by monitoring the decreasing aldehyde concentration over time.

We initiated our studies on a model reaction using terephthalaldehyde as the monomer (**M1**), aiming to assess its polymerization potential and optimize reaction conditions. To drive the photopolymerization through consecutive light‐induced pinacol couplings between the aldehyde functionalities, we employed tertiary amines, which facilitate single‐electron transfer (SET) to the excited‐state aldehyde monomer upon light illumination (**Figure** **2**a).^[^ ^28^ ^]^ Subsequent protonation generates reactive ketyl radicals, which then combine to form the desired pinacol coupling linkage. After screening various reaction conditions (**Table** **1**), we achieved optimized photopolymerization using 0.74 mmol of **M1**, 2 molar equivalents of diisopropylethylamine (DIPEA) as the tertiary amine, dimethylformamide (DMF) as the solvent, and a 365 nm LED for 18 h under an inert argon atmosphere. The final polypinacol **P1** was precipitated from acetonitrile (MeCN) and isolated in 86% yield. ¹H‐NMR spectroscopy confirmed the successful formation of 1,2‐diol species along the polymer backbone, as indicated by ─OH signals at 4.5 ppm and ─CH signals at 5.0–5.5 ppm (Figure 2b). The latter appears as two distinct signals of approximately equal intensity, corresponding to the *meso* and *dl* configurations, suggesting no stereochemical preference in the photopolymerization process. Furthermore, the aldehyde end‐group of the polymer was observed at 9.9 ppm. The molecular weight *M*_w_ of **P1** reached a value of 10,200 g/mol and a dispersity (*Đ*) of 1.75, as determined by gel permeation chromatography (GPC) in DMF (Table 1, entry 1, Figure 2c).

**Table 1:** Optimization of the photopolymerization reaction conditions.

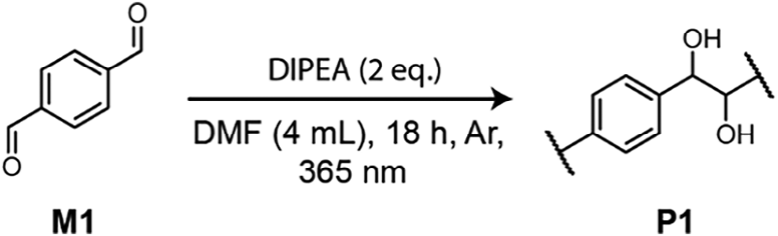					
Entry	Deviation from Standard Conditions^a)^	Yield^b)^ [%]	*M*_w_ ^c)^ [g moL^−1^]	*M*_n_ ^c)^ [g moL^−1^]	*Đ* ^c)^
1	None	86	10,200	5,830	1.75
2	in MeCN	28	19,000	8,080	2.34
3	in MeCN/H_2_O	39	5,700	3,440	1.65
4	in DMSO	53	4,800	3,300	1.45
5	in Me‐THF	5	8,860	5,900	1.50
6	in EtOAc	21	9,940	4,770	2.08
7	in IPA	40	13,650	7300	1.87
8	2 mL of DMF	81	8,100	4,870	1.66
9	6 mL of DMF	35	6,400	4,180	1.53
10	Triethylamine	13	4,040	2,650	1.52
11	DABCO	35	2,130	1,320	1.61
12	Sparteine	95	3,240	2,420	1.33
13	1.0 eq. DIPEA	27	5,540	3,410	1.62
14	0.5 eq. DIPEA	26	4,640	2,950	1.57
15	3.0 eq. DIPEA	28	5,680	3,590	1.58
16	390 nm LED	39	5,340	3,620	1.47
17	No light	0	–	–	–
18	Air	22	7,000	4,700	1.49

^a)^ Standard reaction conditions: Terephthalaldehyde (0.74 mmol), DIPEA (1.49 mmol), 4.0 mL of degassed DMF, 365 nm LED, 18 h;

^b)^ Determined based on isolated compound;

^c)^ Determined by GPC in DMF calibrated with PMMA standard.

Screening the reaction conditions showed that the solvent selection plays a critical role in the photopolymerization. Replacing DMF with other common polar solvents resulted in a significant decrease in both product yield and molecular weight due to the limited solubility and premature precipitation of **P1** in these media (entries 2–7, Figure S1, Supporting Information). Although the usage of MeCN resulted in higher molecular weight of 19 000 g moL^−1^, DMF offered a higher yield and keeps the growing polymer in solution, making it a more favorable solvent overall. Given the importance of concentration in light‐driven reactions, we tested varying DMF volumes. Using higher concentration slightly reduced yield and molecular weight (entry 8), while further dilution drastically lowered them to 35% and 6,400 g moL^−1^, respectively (entry 9). This highlights the delicate balance between light penetration, which improves at lower concentrations, and radical recombination, which is more favorable at higher concentrations. We also explored triethylamine and 1,4‐diazabicyclo[2.2.2]octane (DABCO) as alternative electron donors, but both led to a significant reduction in yield and molecular weight (entries 10–11). This may be attributed to the lower stability of the secondary carbon radical formed after the initial SET and protonation event, compared to the more stable tertiary carbon radical generated with DIPEA. Using sparteine as a tertiary amine resulted in a lower molecular weight (3,160 g moL^−1^), although a high yield (95%) was achieved (entry 12). Both decreasing and increasing the DIPEA amount led to severe reduction in both yield and molecular weight (entries 13–15). Changing the wavelength to 390 nm resulted in a reduction of yield (39%) and molecular weight (5,340 g moL^−1^, entry 16). Control experiments clarified the effects of light and oxygen on the reaction. Performing the reaction in the absence of light resulted in no product formation, highlighting the essential role of light in the polymerization via pinacol coupling (entry 17). Conducting the reaction without argon resulted in polymerization with a molecular weight of 7,000 g moL^−1^; however, the yield was only 22%, likely due to quenching of ketyl radical by oxygen (entry 18). Throughout the screening process, the dispersity values of the final polymers remained reasonably low, ranging from 1.33 to 2.34. Furthermore, all polymerizations—except for entry 17—achieved monomer conversions of ≥99%. Nonetheless, low molecular weights and isolated yields were observed in some cases despite the high conversion. This outcome can be attributed to electronic changes that occur during the polymerization of monomer **M1**. Initially, **M1** contains two conjugated electron‐withdrawing aldehyde groups. Upon pinacol coupling, however, one of these groups is converted into an electron‐donating pinacol moiety, significantly altering the electronic environment of the remaining aldehyde. This change affects the photoreactivity of the system: electron‐withdrawing groups favor the formation of the more reactive T₁(n,π^*^) excited state, while electron‐donating groups stabilize the less reactive T₁(π,π^*^) state.^[^ ^35^, ^36^ ^]^ As a result, the growing polymer chain likely exhibits reduced reactivity compared to the unreacted monomer, leading to rapid initial monomer consumption followed by slower propagation. This imbalance may explain the formation of lower molecular weight polymers despite complete monomer conversion.

After completing the optimization studies, we investigated the reaction kinetics of the photopolymerization using ¹H‐NMR spectroscopy. Initially, the monomer concentration in the reaction mixture decreased significantly, with complete consumption of **M1** occurring within 2 h. Additionally, the peak intensities at 4.5 and 5.0–5.5 ppm, corresponding to the polymer backbone (─OH and ─CH groups), increased over time (Figure S2, Supporting Information). By plotting the concentration of aldehyde functionalities in solution against time, we observed that the reaction followed pseudo‐first‐order kinetics with a rate constant of 0.17 h^−^¹ (Figure 2d). Monomer consumption and polymer formation were also monitored using GPC, showing a gradual increase in molecular weight (Figure S3 and Table S1, Supporting Information).

### 2.2 Substrate Scope

**Figure 3:**
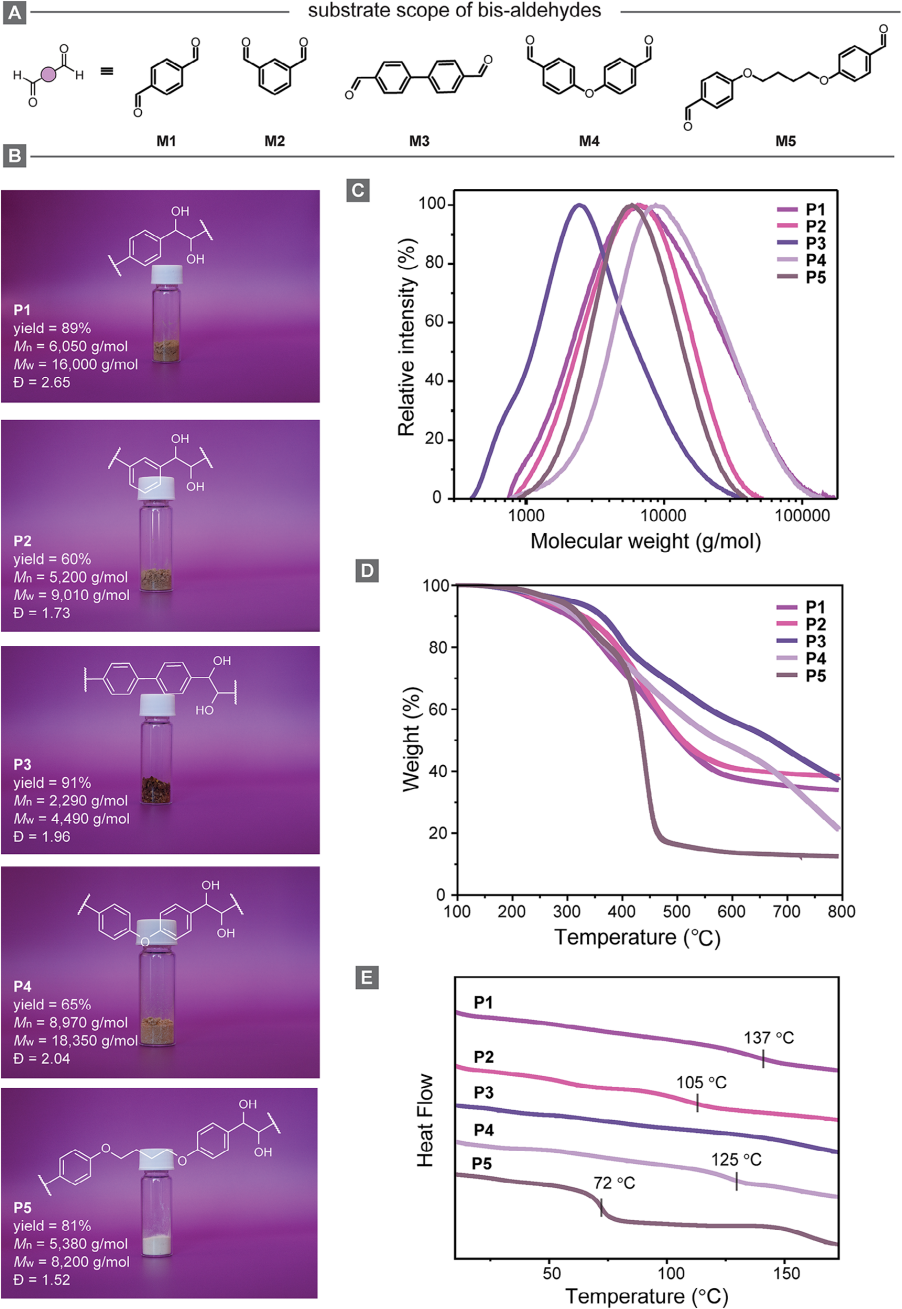
a) Substrate scope of bis‐aldehydes (**M1**–**M5**). b) Photographs of samples of polymers **P1**–**P5**, including yield, *M*_n_, *M*_w_, and dispersity. c) GPC traces, d) TGA curves, ramping from 40 to 800 °C with heating rate of 10 °C min^−1^, and e) DSC curves, indicating *T*_g_ values, of **P1**–**P5**.

To showcase the scalability and versatility of our photopolymerization process, we performed the synthesis of polypinacols using various bis‐aldehyde monomers on a 2 g scale under the optimized conditions (**Figure** **3**a,b). While monomers **M1**–**M4** are commercially available, **M5** was synthesized to enhance monomer flexibility by incorporating an alkyl spacer between the aromatic aldehyde groups (see Supporting Information for details). First, we repeated the preparation of **P1**, confirming that scaling up the reaction does not hinder the photopolymerization. In fact, the larger‐scale synthesis yielded improved results, achieving an 89% yield with a molecular weight of 16 000 g moL^−1^, surpassing the outcomes observed in the optimization studies.

Photopolymerization of the other monomers successfully yielded polypinacols **P2**–**P5**, as confirmed by ¹H‐NMR, ^13^C‐NMR, and FT‐IR spectroscopy (see Figure S4, Supporting Information). For all substrates, complete monomer conversion was observed as evidenced by the disappearance of the aldehyde proton signal of each monomer. GPC measurements revealed *M*_w_ values ranging from 4,490 to 18,350 g moL^−1^ with dispersities between 1.52 and 2.04 (Figure 3c). The relatively low molecular weight for **P3** corresponds only to the soluble fraction of the polymer, as it exhibited limited solubility in DMF, the eluent used for GPC analysis. This solubility issue is likely due to the strong *π*–*π* stacking and the hydrophobic nature of the biphenyl units. We analyzed the thermal properties of the as‐prepared polymers with thermogravimetric analysis (TGA) and differential scanning calorimetry (DSC). TGA analysis demonstrated that the polypinacols **P1**–**P5** have a decomposition temperature at 5% weight loss (*T*_d5%_) in the range of 247 and 306 °C (Figure 3d). Interestingly, polypinacols **P1**–**P3**, which lack ether linkages, exhibited char residues exceeding 30% at 800 °C. In contrast, **P4** and **P5**, which contain ether bonds, experienced more significant thermal degradation. The increased susceptibility of ether bonds to cleavage at elevated temperatures hinders efficient carbonization, resulting in lower char formation. In the DSC measurements, **P1**, **P2**, **P4**, and **P5** displayed glass transition temperatures (*T*_g_) at 137, 105, 125, and 72 °C, respectively (Figure 3e). **P5**, having the most flexible polymer backbone in this series, requires less energy to transition from a rigid glassy state to a more mobile, rubbery state. **P3** did not exhibit a glass transition, most likely because its *T*_g_ exceeds its decomposition temperature.

**Figure 4:**
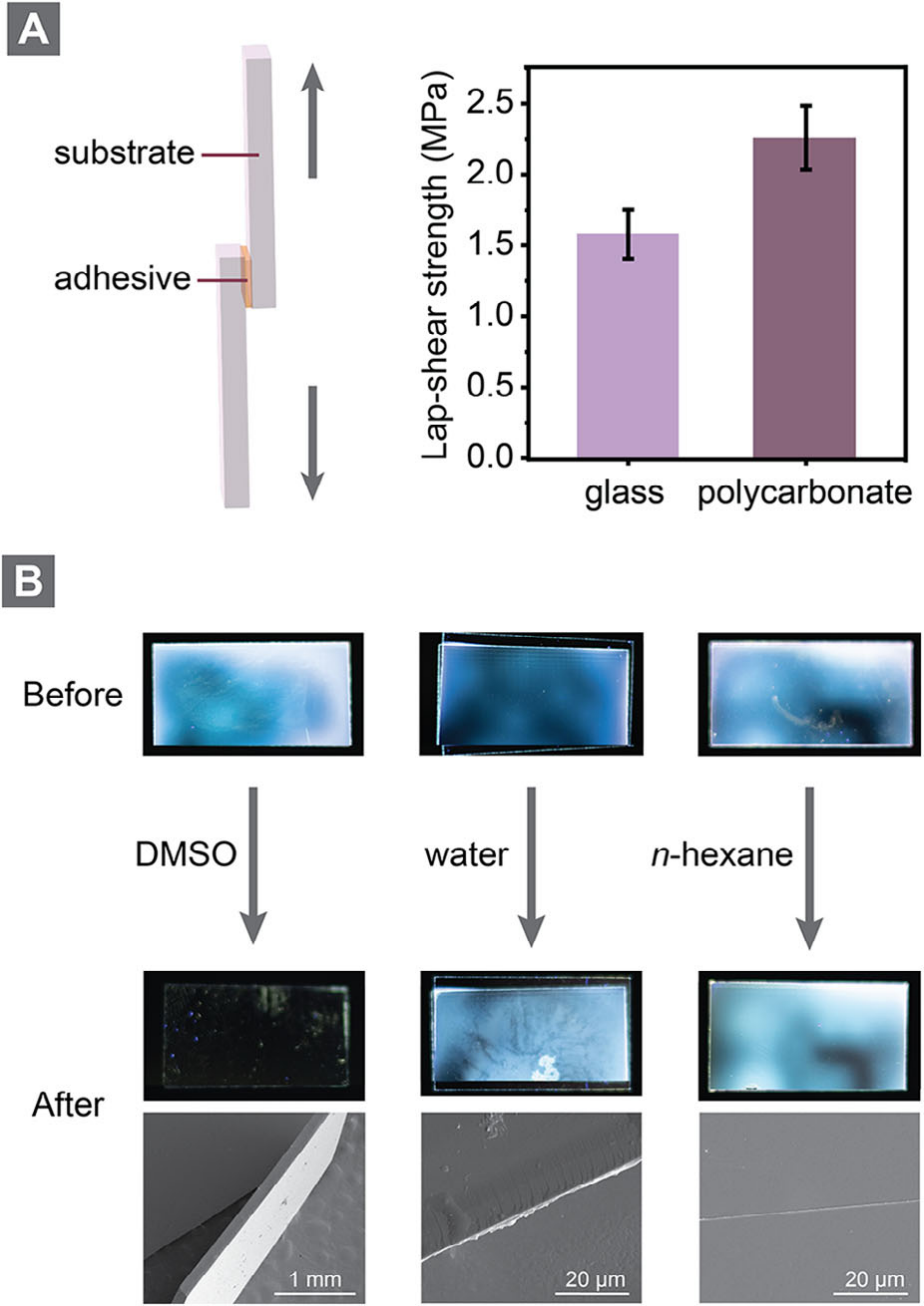
a) Lap‐shear strength of **P5** used as an adhesive between glass and polycarbonate substrates. Error bars represent standard deviations from four independent measurements. b) Fluorescence images of bonded glass substrates before and after debonding tests in DMSO, water, and *n*‐hexane at 40 °C for 24 h. SEM images display a clean glass surface after debonding in DMSO, and intact adhesive layers between the substrate after exposure to water and *n*‐hexane, confirming selective debonding capability.

Given that the synthesized polypinacols are rich in hydroxyl groups capable of forming hydrogen bonds with polar substrates, we explored their potential application as adhesive materials. To evaluate their adhesive performance, **P5** was dissolved in DMF, applied between glass or polycarbonate substrates, and subsequently dried (see Supporting Information for further experimental details). Lap shear testing revealed excellent bonding performance, with shear strengths of 1.6 MPa on glass and 2.0 MPa on polycarbonate (**Figure** **4**a). To investigate the potential for on‐demand debonding, we incorporated a fluorescent dye into the DMF solution before application on glass substrates. After drying, the adhesive displayed excellent substrate wetting and uniform bonding, as evidenced by consistent fluorescence under UV light. Scanning electron microscope (SEM) analysis of the cross section confirmed the presence of a thin, homogeneous adhesive layer between the glass substrates (Figure S5a, Supporting Information). We then conducted solvent‐based debonding tests to assess chemical resistance and reversibility. The adhesive layer remained intact after 24 h of exposure to water or *n*‐hexane at 40 °C, as confirmed by fluorescence imaging and SEM (Figure 4b). However, complete debonding with full recovery of pristine substrates was achieved in DMSO under the same conditions (Figure S5b, Supporting Information), demonstrating the potential for selective solvent‐triggered, reversible adhesion.

### 2.3 Closed‐Loop Photochemical Recycling

After successfully synthesizing polymers via light‐induced pinacol coupling reactions, we aimed to break the newly formed C─C linkages between the monomers using photocatalysis, ultimately regenerating the original monomers and closing the recycling loop. We were inspired by the work of König et al., who demonstrated the photocatalytic cleavage of C─C bonds in small molecule diols using a cerium photocatalyst.^[^ ^34^ ^]^ In the presence of oxygen, Ce(III) is first oxidized to Ce(IV), which then binds to one of the alcohols. The resulting complex is activated by visible light, inducing a SET process from the alcohol to cerium, regenerating the catalyst and forming an alkoxy radical. This radical undergoes a *β*‐scission event, cleaving the C─C bond. Subsequent hydrogen atom transfer then leads to the formation of two separate aldehydes, which, in our case, resembles the generation of the reactive sites of the original monomers (full mechanism shown in Figure S6, Supporting Information).

**Figure 5:**
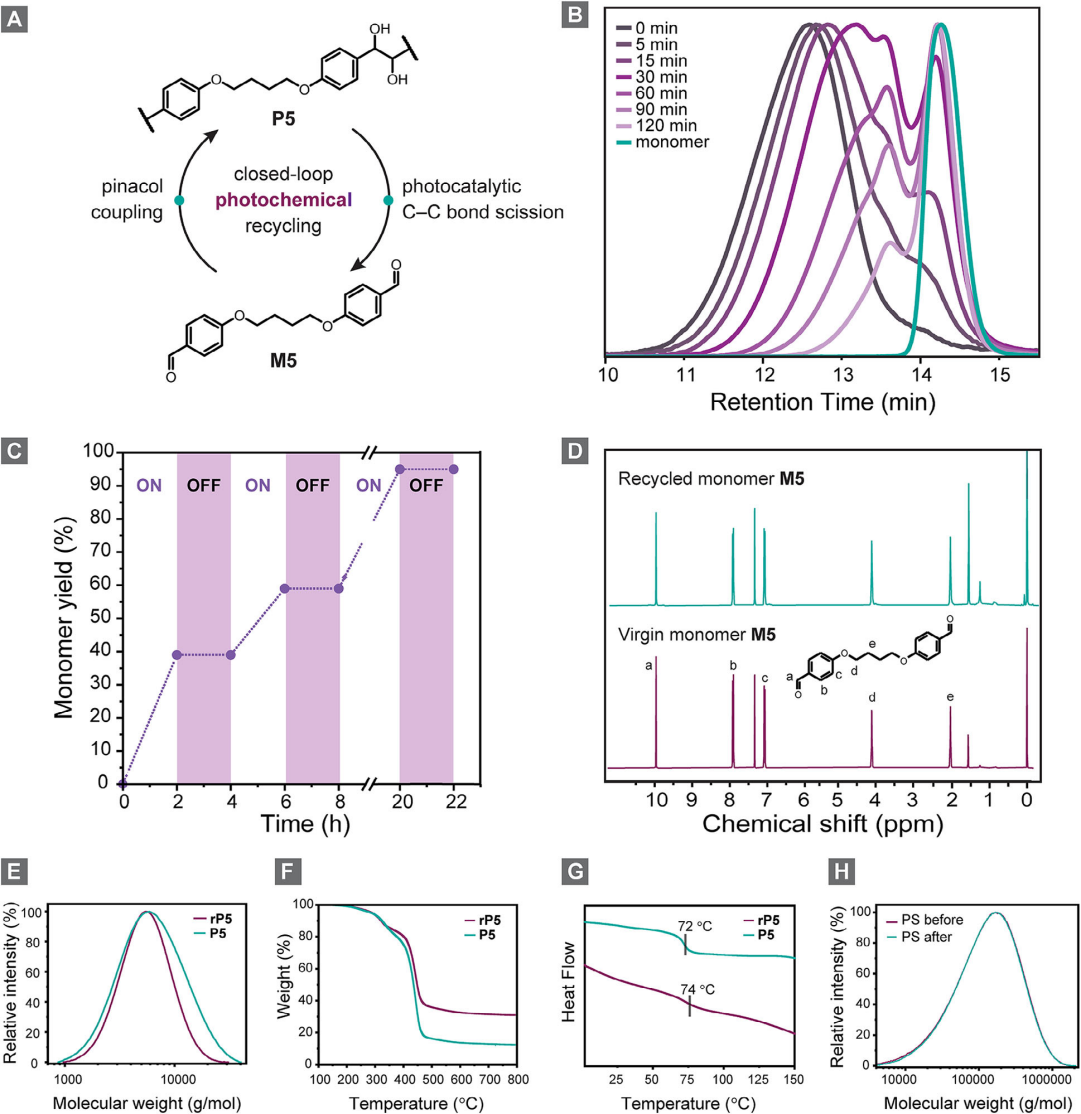
Closed‐loop photochemical recycling. a) Polymer‐to‐monomer recycling scheme for **P5** and **M5**. b) GPC traces measured within the first 120 min of photocatalytic depolymerization. c) Light *on*/*off* cycles during photocatalytic depolymerization. d) ^1^H‐NMR spectra of recycled and original **M5**. e) GPC traces, f) DSC, and g) TGA analysis of recycled and original **P5**. h) GPC measurement of polystyrene before and after the light‐driven recycling process.

We adapted this small molecule transformation to cleave our polypinacols (**Figure** **5**a). To identify optimal reaction conditions (**Table** **2**), we selected **P5** due to its enhanced solubility in DMSO‐d_6_, which allowed us monitor the monomer recovery by ^1^H‐NMR spectroscopy using an internal standard. CeCl₃∙7H₂O (10 mol%) served as the photocatalyst, while tetrabutylammonium chloride (TBACl, 50 mol%) acted as an auxiliary reagent. Exposing the solution to blue light (450 nm) for 52 h in the presence of molecular sieves (MS) to capture residual water yielded 82% of the original monomer **M5** (entry 1). Reducing the catalyst loading to 5 and 2 mol% resulted in slightly lower but still appreciable monomer yields (entries 2–3). Similarly, reducing the TBACl content to 25 or 10 mol%, or omitting it entirely, led to a decrease in yield to 71–61% (entries 4–6). This trend aligns with previous studies in literature, where TBACl promotes the formation of the more catalytically active ((*n*‐Bu₄N)₂Ce(IV)Cl₆) complex.^[^ ^37^ ^]^ To investigate the effect of concentration on the depolymerization process, the solvent volume was reduced to 0.5 mL, which resulted in a decrease in yield to 72% (entry 7). Additional control experiments revealed that molecular sieves are beneficial for achieving high efficiency (entry 8), as water may compete with the polymer's alcohol groups for coordinating with the photocatalyst. Both blue light and the presence of oxygen are strictly required for the reaction to proceed (entries 9–10).

**Table 2:** Optimization of the reaction conditions for photocatalytic depolymerization.

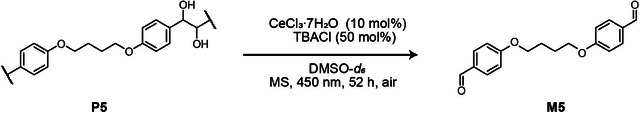		
Entry	Deviation from standard conditions^a)^	Yield^b)^ (%)
1	None	82
2	5 mol% CeCl_3_∙7H_2_O	79
3	2 mol% CeCl_3_∙7H_2_O	71
4	TBACl (25 mol%)	71
5	TBACl (10 mol%)	66
6	No TBACl	61
7	0.5 mL DMSO‐d_6_	72
8	No MS	48
9	No light	0
10	Argon atmosphere	0

^a)^ Standard reaction conditions: **P5** (0.066 mmol), CeCl_3_∙7H_2_O (10 mol%), TBACl (50 mol%), mol sieve powder, 1.0 mL of DMSO‐d_6_, 450 nm LED, 52 h;

^b)^ Determined by ^1^H‐NMR analysis using dimethyl terephthalate as internal standard.

To gain deeper insights into the reaction kinetics, we monitored the photocatalytic depolymerization by periodically extracting aliquots from the reaction mixture and analyzing them via GPC over the first 120 min (Figure 5b; Table S2, Supporting Information). The observed increase in retention time indicates a rapid reduction in the molecular weight of **P5**, suggesting that the depolymerization proceeds through random scission events along the polymer backbone. After 120 min, already a significant amount of short oligomers and monomer was produced. Next, we examined the reaction's dependence on light to rule out unintended thermal depolymerization during the process (Figure 5c). To do so, we conducted multiple light *on*/*off* cycles, alternating between 2 h of illumination and 2 h in darkness. After repeating this cycle twice, we exposed the reaction to continuous light for 16 h, followed by a final 2 h dark phase. ¹H‐NMR spectroscopy revealed that monomer generation occurred exclusively during light exposure, with no detectable increase during dark phases (Figure S7, Supporting Information). These findings confirm that the depolymerization is exclusively light‐driven rather than thermally induced.

We carried out a large‐scale depolymerization of **P5** to regenerate sufficient monomer for reuse in repolymerization under the optimized conditions, demonstrating the feasibility of closed‐loop recycling. After depolymerization, we isolated **M5** in 70% yield with high purity, as demonstrated by ^1^H‐NMR spectroscopy (Figure 5d). The recycled polymer **rP5**, synthesized from the regenerated monomer, exhibited a molecular weight of 6,480 g moL^−1^, a *T*_d5%_ of 288 °C, and a *T*_g_ of 74 °C, all of which were comparable to those of the original polymer **P5** (Figure 5e–g). Lastly, we conducted the depolymerization reaction under the same conditions, with the addition of polystyrene (PS), to explore the potential for orthogonal recycling. PS was specifically chosen due to previous reports of its photocatalytic depolymerization under aerobic conditions.^[^ ^16^, ^17^, ^18^, ^19^, ^20^, ^21^ ^]^ Since our system also operates in the presence of oxygen, we aimed to assess its selectivity. GPC analysis, performed before and after the reaction, confirmed that our system functioned selectively, enabling the recovery of PS without a decrease in its molecular weight (Figure 5h). Furthermore, **M5** was obtained in 81% yield after removal of the PS pieces (Figure S8, Supporting Information).

## 3 Conclusion

Chemical polymer recycling plays a pivotal role in enabling a circular polymer economy by allowing polymeric materials to be selectively broken down and remanufactured, thereby reducing waste and resource consumption. To drive progress in this field, the development of innovative strategies for chemical recycling is essential. In this context, we introduced the first purely photochemical approach to closed‐loop chemical recycling. Our method establishes a light‐driven polymerization–depolymerization cycle specifically tailored for polypinacols, a promising yet largely underexplored class of polymers. Through careful optimization, we unlocked the potential of the pinacol coupling reaction to efficiently stitch together bis‐aldehyde monomers to construct well‐defined linear polymers with adjustable thermal properties. Monomers **M1**–**M5** were successfully converted into their corresponding polymers (**P1**–**P5**) on a 2 g scale using a batch photoreactor. For larger‐scale production and recycling applications, the use of flow photoreactors could provide significant advantages in terms of scalability, efficiency, and process control.^[^ ^38^ ^]^ Due to the strong hydrogen‐bonding capability of the ─OH groups along the polymer backbone, we explored the adhesive properties of **P5** on glass and polycarbonate substrates as a potential application. Lap‐shear tests revealed adhesion strengths of 1.6 and 2.0 MPa, respectively. This performance is comparable with other linear hydroxyl‐rich polymers, such as polyvinyl alcohol.^[^ ^39^ ^]^ DSC and TGA analyses reveal a favorable thermal window for the synthesized polymers, with *T*_g_ values well below the *T*_d5_, indicating good potential for conventional thermal processing methods. Initial compression molding tests were applied for **P5**, but the resulting polymer film showed cracking due to its brittleness. To improve the mechanical integrity, further structural modifications to the polymer backbone will be necessary. By harnessing an earth‐abundant and simple cerium photocatalyst, we achieved selective cleavage of polypinacols under visible light, efficiently regenerating the original monomer. By tolerating the presence of polystyrene, a common commodity plastic, this approach indicates the potential for orthogonal recycling strategies capable of selectively recovering individual polymers from mixed plastic waste streams. The completion of one recycling cycle, yielding a polymer with properties on par with the original one, effectively illustrates the benefits and potential of (photo)chemical recycling.

## Conflict of Interest

The authors declare no conflict of interest.

## Supporting information



Supporting Information

## Data Availability

The data that support the findings of this study are available from the corresponding author upon reasonable request.

## References

[1] G. W. Coates , Y. D. Y. L. Getzler , Nat. Rev. Mater. 2020, 5, 501.

[2] R. Geyer , J. R. Jambeck , K. L. Law , Sci. Adv. 2017, 3, 1700782. 10.1126/sciadv.1700782 PMC5517107 28776036

[3] L. D. Ellis , N. A. Rorrer , K. P. Sullivan , M. Otto , J. E. McGeehan , Y. Román‐Leshkov , N. Wierckx , G. T. Beckham , Nat. Catal. 2021, 4, 539.

[4] A. R. Rahimi , J. M. Garciá , Nat. Rev. Chem. 2017, 1, 0046.

[5] S. D. Anuar Sharuddin , F. Abnisa , W. M. A. Wan Daud , M. K. Aroua , Energy Convers. Manage. 2016, 115, 308.

[6] L. Wimberger , G. Ng , C. Boyer , Nat. Commun. 2024, 15, 2510. 38509090 10.1038/s41467-024-46656-3 PMC10954676

[7] I. M. Irshadeen , S. L. Walden , M. Wegener , V. X. Truong , H. Frisch , J. P. Blinco , C. Barner‐Kowollik , J. Am. Chem. Soc. 2021, 143, 21113. 34859671 10.1021/jacs.1c09419

[8] J. Vanderghinste , S. Das , Synthesis 2022, 54, 3383.

[9] X. Y. Yu , J. R. Chen , W. J. Xiao , Chem. Rev. 2021, 121, 506. 32469528 10.1021/acs.chemrev.0c00030

[10] F. Eisenreich , Angew. Chem., Int. Ed. 2023, 62, 202301303. 10.1002/anie.202301303 37051840

[11] K. Parkatzidis , H. S. Wang , A. Anastasaki , Angew. Chem., Int. Ed. 2024, 63, 202402436. 10.1002/anie.202402436 38466624

[12] C. Zhang , Q. Kang , M. Chu , L. He , J. Chen , Trends Chem 2022, 4, 822.

[13] S. T. Nguyen , E. A. McLoughlin , J. H. Cox , B. P. Fors , R. R. Knowles , J. Am. Chem. Soc. 143, 12268. 10.1021/jacs.1c05330 34333967

[14] S. T. Nguyen , L. R. Fries , J. H. Cox , Y. Ma , B. P. Fors , R. R. Knowles , J. Am. Chem. Soc. 2023, 145, 11151. 37167410 10.1021/jacs.3c00958

[15] S. Gazi , M. Đokić , K. F. Chin , P. R. Ng , H. S. Soo , Adv. Sci. 2019, 6, 1902020. 10.1002/advs.201902020 PMC6918108 31871870

[16] S. Oh , E. E. Stache , J. Am. Chem. Soc. 2022, 144, 5745. 35319868 10.1021/jacs.2c01411

[17] S. Oh , E. E. Stache , ACS Catal. 2023, 13, 10968.

[18] G. Zhang , Z. Zhang , R. Zeng , Chin. J. Chem. 2021, 39, 3225.

[19] M. Wang , J. Wen , Y. Huang , P. Hu , ChemSusChem 2021, 14, 5049. 34510789 10.1002/cssc.202101762

[20] Z. Huang , M. Shanmugam , Z. Liu , A. Brookfield , E. L. Bennett , R. Guan , D. E. Vega Herrera , J. A. Lopez‐Sanchez , A. G. Slater , E. J. L. McInnes , X. Qi , J. Xiao , J. Am. Chem. Soc. 2022, 144, 6532. 35353526 10.1021/jacs.2c01410 PMC9011358

[21] T. Li , A. Vijeta , C. Casadevall , A. S. Gentleman , T. Euser , E. Reisner , ACS Catal. 2022, 12, 8155. 35874621 10.1021/acscatal.2c02292 PMC9295126

[22] G. Ciamician , P. Silber , C. Lichtwirkungen , Ber. Dtsch. Chem. Ges. 1900, 33, 2911.

[23] D. E. Pearson , P. D. Thiemann , J. Polym. Sci. A‐1 Polym. Chem. 1970, 8, 2103.

[24] J. Higgins , A. H. Johannes , J. F. Jones , R. Schultz , D. A. McCombs , C. S. Menon , J. Polym. Sci. 1970, 8, 1987.

[25] D. A. McCombs , C. S. Menon , J. Higgins , J. Polym. Sci. Part A‐1 Polym. Chem. 1971, 9, 1261.

[26] Z. Qiu , H. D. M. Pham , J. Li , C. C. Li , D. J. Castillo‐Pazos , R. Z. Khaliullin , C. J. Li , Chem. Sci. 2019, 10, 10937. 32190250 10.1039/c9sc03737c PMC7066673

[27] Q. Shen , K. Cao , X. Chen , X. Li , N. Zhang , Y. B. Miao , J. Li , Green Chem. 2023, 25, 9665.

[28] Y. Yan , G. Li , J. Ma , C. Wang , J. Xiao , D. Xue , Green Chem. 2023, 25, 4129.

[29] M. Nakajima , E. Fava , S. Loescher , Z. Jiang , M. Rueping , Angew. Chem., Int. Ed. 2015, 127, 8952. 10.1002/anie.201501556 26082970

[30] R. Naumann , M. Goez , Green Chem. 2019, 21, 4470.

[31] F. Calogero , G. Magagnano , S. Potenti , F. Pasca , A. Fermi , A. Gualandi , P. Ceroni , G. Bergamini , P. G. Cozzi , Chem. Sci. 2022, 13, 5973. 35685797 10.1039/d2sc00800a PMC9132033

[32] E. Pinosa , Y. Gelato , F. Calogero , M. M. Moscogiuri , A. Gualandi , A. Fermi , P. Ceroni , P. G. Cozzi , Adv. Synth. Catal. 2024, 366, 798.

[33] R. Wang , M. Ma , X. Gong , X. Fan , P. J. Walsh , Org. Lett. 2019, 21, 27. 30484653 10.1021/acs.orglett.8b03394

[34] J. Schwarz , B. König , Chem. Commun. 2019, 55, 486. 10.1039/c8cc09208g 30548043

[35] P. J. Wagner , R. J. Truman , J. C. Scaiano , J. Am. Chem. Soc. 1985, 107, 7093.

[36] W. J. Leigh , E. C. Lathioor , M. J. St Pierre , J. Am. Chem. Soc. 1996, 118, 12339.

[37] A. Hu , J. J. Guo , H. Pan , Z. Zuo , Science 2018, 361, 668. 30049785 10.1126/science.aat9750

[38] L. Buglioni , F. Raymenants , A. Slattery , S. D. A. Zondag , T. Noël , Chem. Rev. 2022, 122, 2752. 34375082 10.1021/acs.chemrev.1c00332 PMC8796205

[39] H. Pingan , J. Mengjun , Z. Yanyan , H. Ling , RSC Adv. 2017, 7, 2450.

