# Supplementary material for: A Light‐Driven Closed‐Loop Chemical Recycling System for Polypinacols
Source: Adv Mater. 2025 Jun 29;37(38):2506733. doi: 10.1002/adma.202506733 (PMC12464638; doi:10.1002/adma.202506733)
Supplement: Supplementary file 1 — Supporting Information [file ADMA-37-2506733-s001.pdf]

ADVANCED MATERIALS

Supporting Information

for *Adv. Mater.*, DOI 10.1002/adma.202506733

A Light-Driven Closed-Loop Chemical Recycling System for Polypinacols

*Ahsen Sare Yalin, Patrick Schara, Željko Tomović and Fabian Eisenreich**

Supporting Information

A Light-Driven Closed-Loop Chemical Recycling System for Polypinacols

*Ahsen Sare Yalin, Patrick Schara, Željko Tomović, and Fabian Eisenreich**

A. S. Yalin, P. Schara, Prof. Dr. Ž. Tomović, Dr. F. Eisenreich
Department of Chemical Engineering and Chemistry, Institute for Complex Molecular Systems (ICMS), Eindhoven University of Technology, 5600 MB Eindhoven, The Netherlands

E-mail: f.r.eisenreich@tue.nl

Contents

1 Experimental section ... 3
1.1 Materials ... 3
1.2 Equipment ... 3
1.3 Synthesis of monomer ... 4
1.4 Optimization of polymerization through photoinduced pinacol coupling ... 4
1.5 Kinetic study ... 6
1.6 Synthesis of polymers ... 8
1.9 Lap Shear Measurements for Adhesive Applications ... 12
1.10 Optimization of depolymerization through photocatalysis ... 12
1.11 Kinetic study for depolymerization ... 13
1.12 Light *on*/*off* experiment ... 14
1.13 Closed-Loop Recycling ... 14
References ... 16
Appendix ... 17

1 Experimental section

1.1 Materials

Terephthalaldehyde, (+)-sparteine, and sodium hydroxide, methyl-THF were purchased from TCI Europe. Isophthalaldehyde, [1,1'-biphenyl]-4,4'-dicarbaldehyde, 4,4'-oxydibenzaldehyde, and 1-bromo-2-(2- bromoethoxy)ethane, 2,5-Bis(5-(tert-butyl) benzo[d]oxazol-2-yl)thiophene, was used as a fluorescent marker were purchased from BLD Pharmatech Gmbh. 4-hydroxybenzaldehyde, potassium carbonate, 1,4-dibromobutane, *N*,*N*-diisopropylethyl-amine, triethylamine, 1,4-diazabicyclo[2.2.2]octane (DABCO), dimethyl terephthalate, cerium(III) chloride heptahydrate, tetrabutylammonium chloride were purchased from Merck Life Science NV. Chloroform-*d* (99.8%), dimethylsulfoxide-d_6 (99.9%) were purchased from Cambridge Isotope Laboratories. Acetonitrile, dimethylsulfoxide (DMSO), ethylacetate, isopropanol (IPA), *N*,*N*-dimethylformamide (DMF) were purchased from Biosolve B.V.

1.2 Equipment

^{1}H and ^{13}C nuclear magnetic resonance (NMR) spectroscopy was conducted using a Bruker Ultra Shield 400 MHz at room temperature. Gel permeation chromatography (GPC) measurements of diol-based polymers were performed on LC-250C (Shimazu) equipped with two PSS GRAM liner 10 μm columns and a refractive index (RI) detector. The polymers were dissolved in DMF with 0.1 M LiBr (1.5 mg/mL) and the polymer solutions were filtered through a 0.2 μm PTFE filter before analysis. DMF was used as the eluent with a flow rate of 1 mL/min and the GPC traces were calibrated with PMMA as standard to calculate the molecular weight and dispersities. Fourier transformation infrared spectroscopy (FT-IR) was carried out in attenuated total reflection (ATR) mode on a Thermo Scientific NICOLET iS20 FTIR Spectrometer. Thermogravimetric analysis (TGA) was performed using TGA550 (TA instruments). Determined amount of polymers (3–5 mg) were put on a platinum pan for measurement. The samples were first equilibrated at 100 °C for 30 min, followed by heating from 100 to 800 °C with a rate of 10 °C/min under a nitrogen atmosphere. Differential scanning calorimetry (DSC) was performed on TA Q2000 (TA instruments). The samples (3–5 mg) were measured in an aluminum pan. The experiments were carried out from room temperature to 200 °C at a rate of 5 °C/min under argon atmosphere. Glass transition temperatures (T_g) were determined by taking the midpoint of the reversible endotherm of the second heating. A Penn PhD Photoreactor M2 with two different LEDs, 365 nm and 450 nm, was utilized to conduct the photoreactions. Lap shear tests were conducted on Zwick/Roell Intelligent at a strain rate of 0.5 mm/min, utilizing a pre-force of 1 N. The polycarbonate test bars were bonded together with a specimen overlap of 2.5 × 2.5 cm^2. The glass substrates were bonded together with a specimen overlap of 1.0 × 2.5 cm^2. The lap shear values reported are an average of at least four different samples.

1.3 Synthesis of monomer

1.3.1 Synthesis of 4,4′-(butane-1,4-diylbis(oxy))dibenzaldehyde

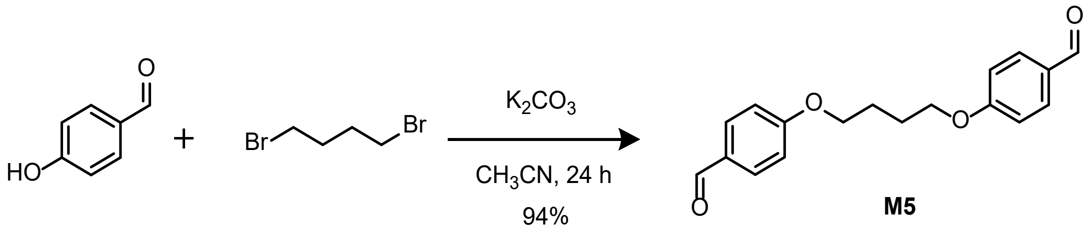

Monomer **M5** was synthesized based on literature.[1]

A three-necked round bottom flask was charged with 4-hydroxybenzaldehyde (81.8 mmol, 2.4 equiv.), anhydrous K_2CO_3 (81.80 mmol, 2.4 equiv.), and CH_3CN (75 mL). The mixture was stirred under an inert atmosphere of argon for 30–45 minutes. Subsequently, 1,4-dibromobutane (34.12 mmol, 1.0 equiv.) was added. The reaction was carried out for 24 h under reflux. Upon completion, the reaction mixture was poured into water to remove salts. The resulting precipitate was filtered, then dissolved in CH_2Cl_2 and washed with a NaOH (1M) solution and distilled water. The organic phase was dried over $MgSO_4$ and the solvent was removed under reduced pressure using a rotary evaporator. After overnight drying, product was obtained as white-powder with a yield of 94%. ^{1}H NMR (400 MHz, DMSO-*d*$_6$): δ = 9.87 (s, 2H, CHO), 7.89–7.83 (m, 4H, Ar-H), 7.16–7.10 (m, 4H, Ar-H), 4.17 (h, J = 3.3 Hz, 4H), 1.92 (h, J = 2.8 Hz, 4H) ppm.

1.4 Optimization of polymerization through photoinduced pinacol coupling

100 mg (0.745 mmol) of terephthalaldehyde, a determined amount of DIPEA, and DMF were added into a round-bottom glass tube equipped with a stirring bar. The reaction mixture was degassed under argon, and the glass tube was located in the photoreactor equipped with a 365 nm LED. Light illumination was applied for 18 h. Afterwards, the solvent was removed under reduced pressure using a rotary evaporator. The resulting crude product was dissolved in 0.5 mL of DMF and precipitated into 5–6 mL of acetonitrile. The precipitate was collected after centrifugation, washed with MeCN and EtOH, and dried overnight in vacuum oven. The final polymer was characterized by ^{1}H-NMR spectroscopy and GPC. The conversion of **M1** was determined by monitoring the disappearance of the aldehyde and aromatic proton signals at 10.14 and 8.12 ppm, respectively, in the ^{1}H-NMR spectrum of the mixtures.

1.4.1 GPC analysis of polymers generated during optimization studies

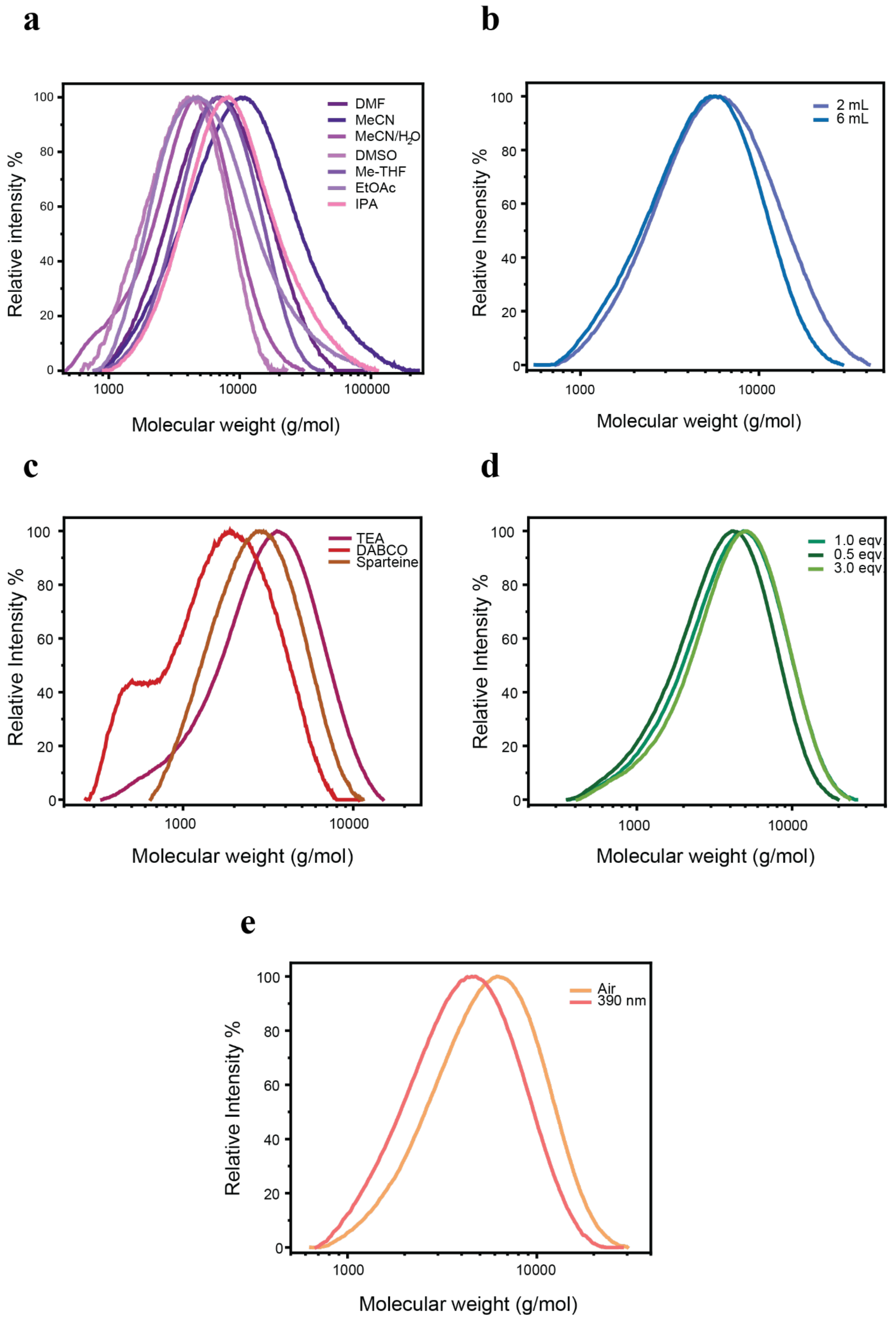

Figure S1. GPC traces of **P1** for the reactions conducted in a) different solvents, b) different concentrations, c) different amines, d) different ratios of DIPEA, e) different reaction conditions. The results are given in Table 1 in the manuscript.

1.5 Kinetic study

Terephthalaldehyde (750 mg, 5.59 mmol, 1 equiv.), DIPEA (1.95 mL, 11.18 mmol, 2 equiv.), and 30 mL of DMF (HPLC grade) were added into a 40 mL vial and degassed under argon for a few minutes. The reaction mixture was then placed inside the photoreactor, and light illumination at 365 nm was initiated. To monitor the reaction progress, samples were taken from the solution to analyze at specified time intervals (every 30 min for the first hour, then hourly). The samples were analyzed by ^{1}H NMR spectroscopy to determine monomer conversion, which was calculated based on the ratio of the aldehyde signals (from both the monomer and polymer chain ends) to the –OH signal, as shown in Figure S2. The natural logarithm of $[\text{aldehyde}]_0/[\text{aldehyde}]$ was plotted over time to assess the reaction kinetics (Figure 2D). In addition, GPC measurements were conducted on crude samples collected at various time points to monitor the evolution of polymer molecular weight. These measurements, which show lower molecular weights due to the crude nature of the samples, reveal the expected increase in retention time/molecular weight over time, as presented in Table S1 and Figure S3.

Table S1. Change in molecular weight and its distribution over time

Time (h)	M_w (g/mol)[a]	M_n (g/mol)[a]	Đ[a]
1	940	840	1.11
2	1,040	890	1.17
4	1,190	960	1.22
6	1,400	1,070	1.30
8	1,780	1,230	1.44
10	2,220	1,410	1.60
12	2,470	1,490	1.65
14	2,950	1,640	1.80
16	3,320	1,740	1.91
18	3,670	1,800	2.03
24	4,600	2,000	2.30

[a)]Determined by GPC in DMF calibrated with PMMA standard for crude samples.

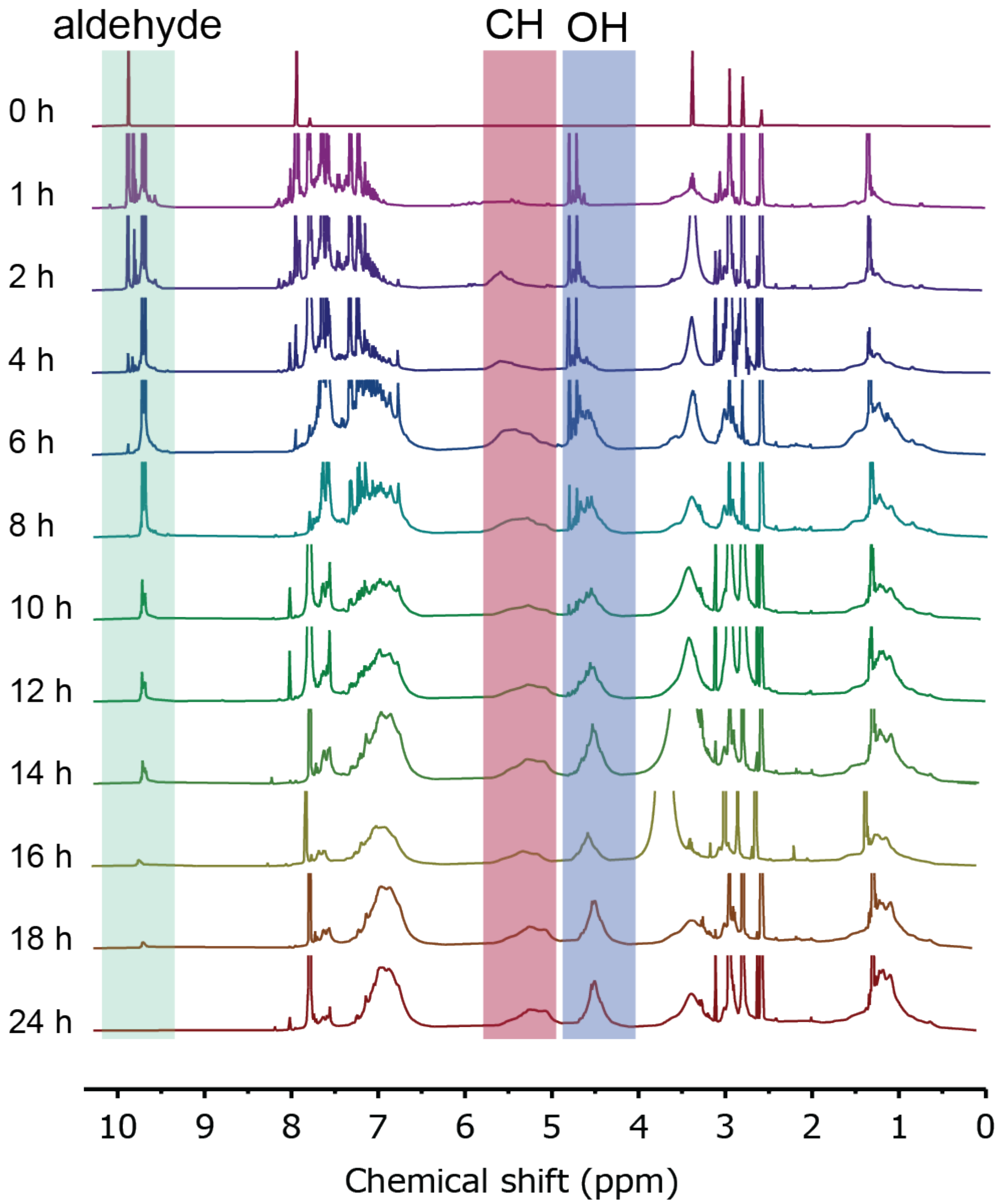

Figure S2. ^{1}H NMR spectra (400 MHz, DMSO-d_6) recorded at various time points during the kinetic study of the photopolymerization of **M1** to form **P1**.

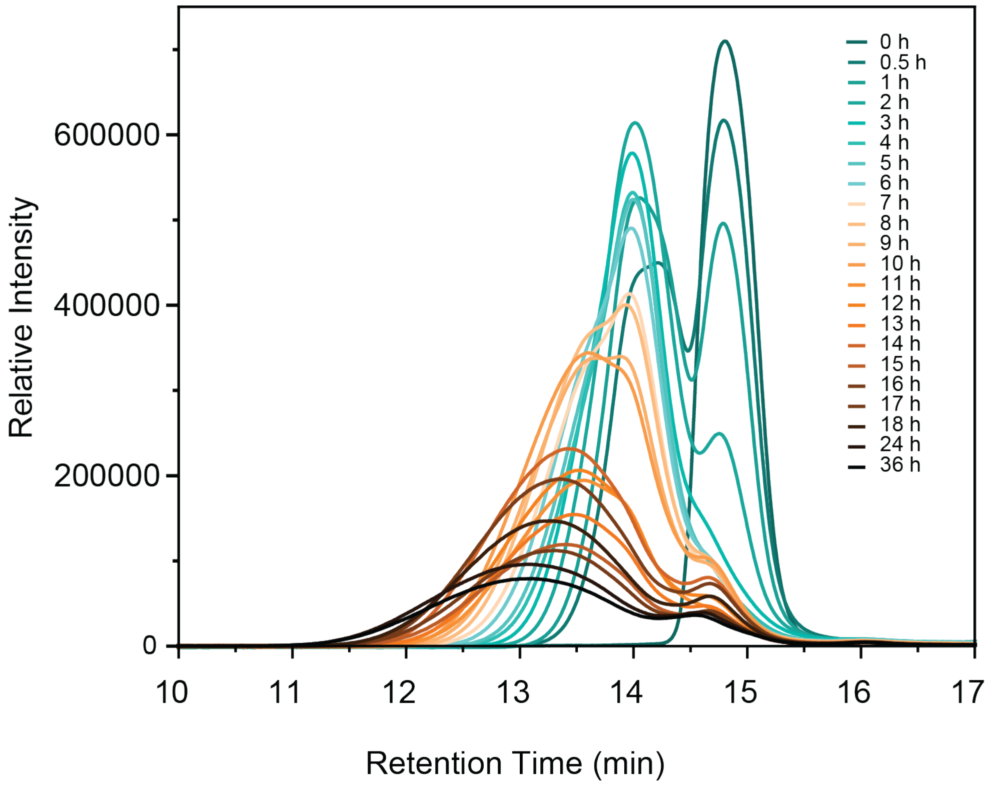

Figure S3. GPC traces of crude **P1** polymer samples collected at various time points during the photopolymerization kinetic study.

1.6 Synthesis of polymers

1.6.1 Synthesis of polypinacols

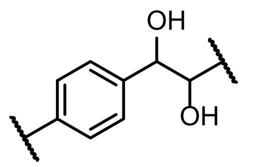

P1

2 g (14.91 mmol, 1.0 equiv.) of terephthalaldehyde (**M1**), 5.20 mL (29.82 mmol, 2.0 eqv.) of DIPEA, and 30 mL of DMF was added in a 40 mL vial, and degassed under argon. The light illumination was started with placing the vial inside the photoreactor for 48 h. After the reaction, excess DMF was removed under reduced pressure. The resulting product was dissolved in a few milliliters of DMF and precipitated into diethylether (DE). The second precipitation was performed into acetonitrile (MeCN). A fine powder precipitate was formed, which was collected by filtration. The precipitate was washed with MeCN. The final polymer **P1** was dried in vacuum oven for overnight and obtained in 89% yield. ^{1}H NMR (DMSO-*d*$_6$): δ = 7.49–6.53 (4H, Ar-H), 5.69–4.85 (*meso*:*dl* 1:1, 2H, C-H), 4.70–4.20 (2H, O-H) ppm. ^{13}C NMR (DMSO-*d*$_6$): δ = 193.33 (H-C=O, end group), 140.70 (Ar-C, end group), 130.54–122.86 (Ar-C, *meso* & *dl*), 77.09 (C-OH, *meso* & *dl*) ppm.

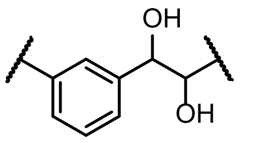

P2

2 g (14.91 mmol, 1.0 equiv.) of isophtalaldehyde (**M2**), 5.20 mL (29.82 mmol, 2.0 equiv.) of DIPEA, and 30 mL of DMF were added to a 40 mL glass vial. After degassing under argon, the vial was located in the photoreactor equipped with a 365 nm LED and light illumination was initiated for 48 h. Afterwards, excess DMF was removed under reduced pressure and the crude product was dissolved in a few mL of DMF followed by precipitation in DE. The collected polymer sample was dissolved again in DMF and precipitated from MeCN. The polymer was collected via vacuum filtration and remains were washed with MeCN. The final polymer **P2** was obtained in 60% yield. ^{1}H NMR (DMSO-d$_6$): δ = 8.10–6.41 (4H, Ar-H), 5.59–4.85 (2H, C-H), 4.77–4.15 (2H, O-H) ppm. ^{13}C NMR (DMSO-*d*$_6$): δ = 193.63 (H-C=O, end group), 142.70 (Ar-C, end group), 129.87–122.81 (Ar-C, *meso & dl*), 77.65 (C-OH, *meso & dl*) ppm.

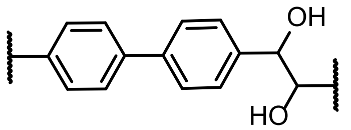

P3

2 g (9.51 mmol, 1.0 equiv.) of [1,1'-biphenyl]-4,4'-dicarbaldehyde (**M3**), 3.31 mL (19.03 mmol, 2.0 equiv.) of DIPEA, and 30 mL of DMF were added to a 40 mL glass vial. After degassing under argon, the vial was located in the photoreactor equipped with a 365 nm LED and light illumination was initiated for 48 h. Upon completion, excess DMF was removed under reduced pressure and the crude product was dissolved in DMF before precipitating into MeCN. The precipitate was collected via vacuum filtration, washed with MeCN and EtOH followed with drying in a vacuum oven overnight. The final polymer **P3** was obtained in 91% yield. ^{1}H NMR (DMSO-*d*$_6$): δ = 7.79–7.11 (8H, Ar-H), 5.53–5.22 (2H, C-H), 4.75–4.54 (2H, O-H) ppm. ^{13}C NMR spectrum was not recorded due to the low solubility of **P3**.

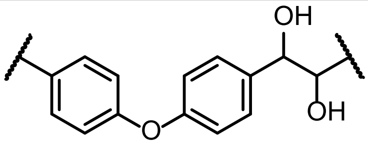

P4

2 g (8.84 mmol, 1.0 equiv.) of 4,4'-oxydibenzaldehyde (**M4**), 3.08 mL (17.68 mmol, 2.0 equiv.) of DIPEA, and 30 mL of DMF were added to a 40 mL glass vial. After degassing under argon, the vial was located in the photoreactor equipped with a 365 nm LED and light illumination was initiated for 48 h. Upon completion, excess DMF was removed under reduced pressure and the crude product was dissolved in DMF before precipitating into MeCN. After vacuum filtration for collecting of the polymer, sample was dried overnight in the vacuum oven. The final polymer **P4** was obtained in 65% yield. ^{1}H NMR (DMSO-*d*$_6$): δ = 7.38–6.70 (8H, Ar-H), 5.35 (*meso*, 1H, C-H), 5.19 (*dl*, 1H, C-H), 4.63–4.41 (2H, O-H) ppm. ^{13}C NMR (DMSO-*d*$_6$): δ = 155.98 (s, Ar-C-O), 138.69 (Ar-C-CHOH, *meso*), 137.64 (Ar-C-CHOH, *dl*), 129.20 (Ar-C-CH, *meso & dl*), 117.89 (m, Ar-C-C-O), 77.56 (s, C-OH, *meso*), 76.93 (C-OH, *dl*) ppm.

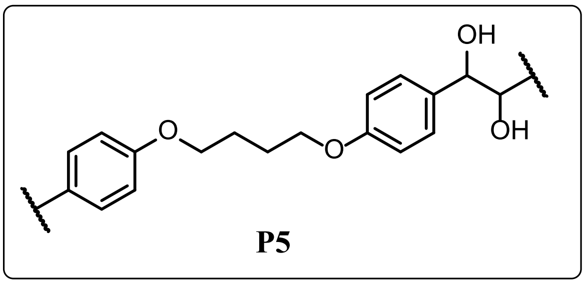

2 g (6.70 mmol, 1 equiv.) of monomer **M5**, 2.34 mL (13.41 mmol, 2.0 equiv.) of DIPEA, and 30 mL of DMF were added to a 40 mL glass vial. After degassing under argon, the vial was located in the photoreactor equipped with a 365 nm LED and light illumination was initiated for 48 h. Upon completion, excess DMF was removed under reduced pressure and the crude product was dissolved in DMF before precipitating into MeCN. The precipitate was collected with vacuum filtration, washed with MeCN, and dried overnight in the vacuum oven. The final polymer **P5** was obtained in 81% yield. ^{1}H NMR (DMSO-d_6): δ = 7.23–6.64 (8H, Ar-H), 5.22 (*meso*, 1H, C-H), 5.03 (*dl*, 1H, C-H), 4.47 (2H, O-H), 4.40–3.83 (4H, --OCH_2), 1.92–1.71 (4H, CH_2) ppm. ^{13}C NMR (DMSO-d_6): δ = 157.84 (Ar-C-O), 135.77 (Ar-C-CHOH, *meso*), 134.75 (Ar-C-CHOH, *dl*), 128.80 (Ar-C-CH, *meso* & *dl*), 113.07 (Ar-C-C-O), 77.84 (C-OH, *meso*), 77.07 (C-OH, *dl*), 67.45 (C-CH_2-O), 25.97 (CH_2) ppm.

1.7 Characterization of linear polymers

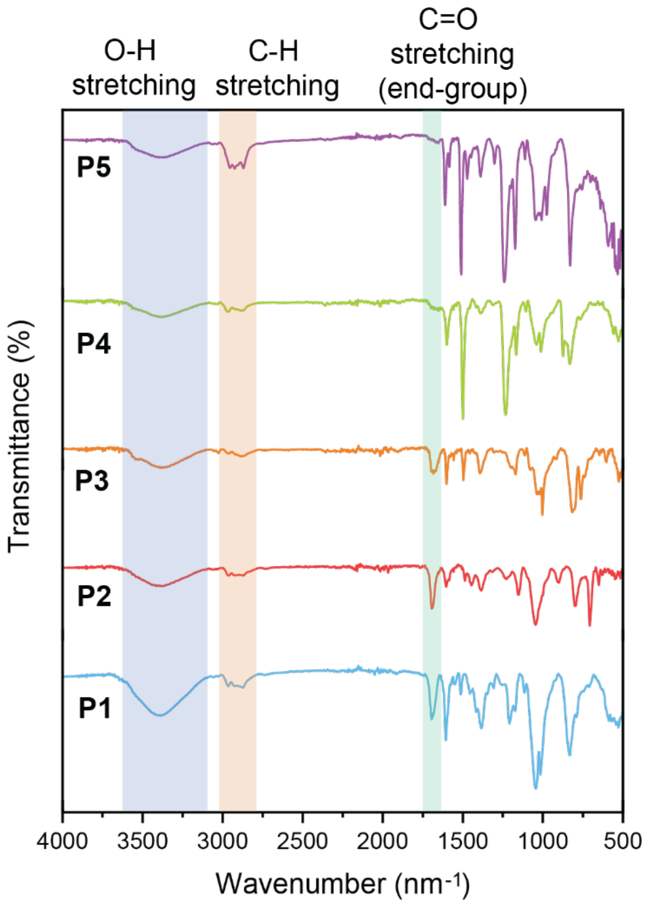

Figure S4. FTIR spectra of polypinacols **P1–P5**.

1.8 Debonding Experiments for Adhesive Applications

For on-demand debonding experiments, 300 mg of **P5** was dissolved in 3 mL of DMF. 1 mg of 2,5-bis(5-(*tert*-butyl)benzo[d]oxazol-2-yl)thiophene as fluorescence marker was added to the previously prepared solution of **P5**. Three drops of the resulting solution were applied between glass slides (1.5 × 1.5 cm^2), which were then bonded. The adhesive assemblies were placed in a nitrogen oven at 100 °C for 24 h. Subsequently, each bonded sample was placed in a 25 mL vial containing 10 mL of either DMSO, water, or *n*-hexane. Photographs of each sample were taken prior to debonding. The vials were then placed on a heating plate maintained at 40 °C overnight. After solvent exposure, the samples were removed, dried, and inspected. Debonding was observed only for the sample immersed in DMSO, while the others remained intact. Photographs were taken after debonding tests. Scanning electron microscopy (SEM) analysis was performed on the cross-sections of all samples to assess the integrity and morphology of the adhesive layer (Figure 4b, S5).

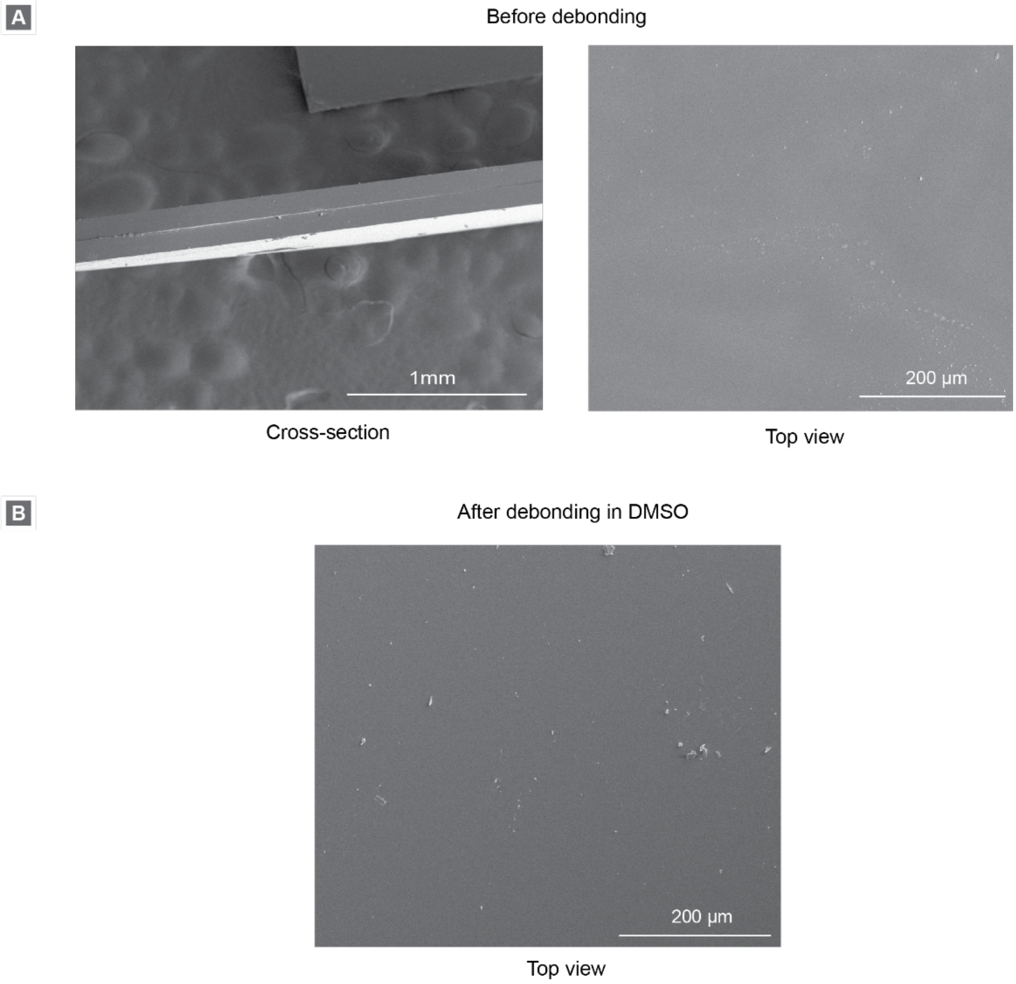

Figure S5. a) SEM images of adhesive between (left) glass slides, cross-section, and (right) top view. b) SEM image of glass slide after debonding, top view, showing no residue of **P5** on the surface.

1.9 Lap Shear Measurements for Adhesive Applications

For lap-shear measurements with glass substrates, 300 mg of **P5** was dissolved in 3 mL of DMF, and 2 drops were applied on a glass slides. The glass substrates were bonded together with a specimen overlap of 1.0 × 2.5 cm^2, a 300 g weight was then placed on top of the adhesive assembly ensuring sufficient and homogeneous bonding between the two substrates. The adhesive assembly was then placed in a 100 °C nitrogen oven for 24 h. Lap shear tests were conducted on Zwick/Roell Intelligent at a strain rate of 0.5 mm/min, utilizing a pre-force of 1 N.

For lap-shear measurements with polycarbonate substrates, 300 mg of **P5** was dissolved in 3 mL of DMF, and 5 drops were applied on a polycarbonate test bar. The polycarbonate test bars were bonded together with a specimen overlap of 2.5 × 2.5 cm^2, a 300 g weight was then placed on top of the adhesive assembly ensuring sufficient and homogeneous bonding between the two substrates. The adhesive assembly was then placed in a 100 °C nitrogen oven for 24 h. Lap shear tests were conducted on Zwick/Roell Intelligent at a strain rate of 0.5 mm/min, utilizing a pre-force of 1 N.

1.10 Optimization of depolymerization through photocatalysis

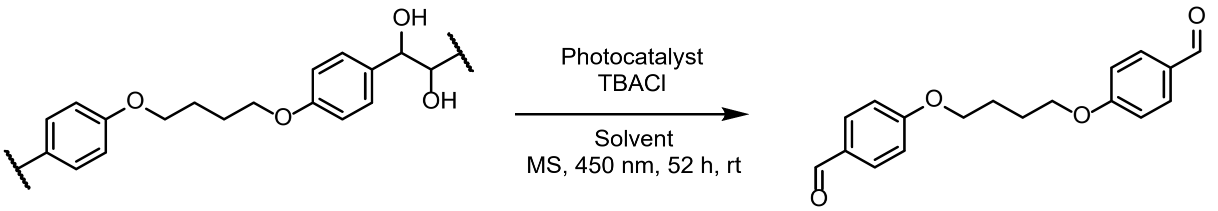

20 mg (0.066 mmol, 1.0 equiv. of repeating unit) of polymer, 12.9 mg (0.066 mmol, 1.0 equiv.) of dimethyl terephthalate as an internal standard, the corresponding amount of tetrabutylammonium chloride (TBACl), the determined amount of photocatalyst, molecular sieve (MS), and 1.0 mL of solvent were added in a 1.0 mL vial equipped with a stirring bar. Locating the vial in the photoreactor equipped with 450 nm LED was followed by light irradiation for 52 h. After solvent removal, if necessary, NMR samples were prepared in DMSO-d_6. The NMR yield was calculated by comparing the aromatic peak of the internal standard and the aldehyde peak of the product **M5**.

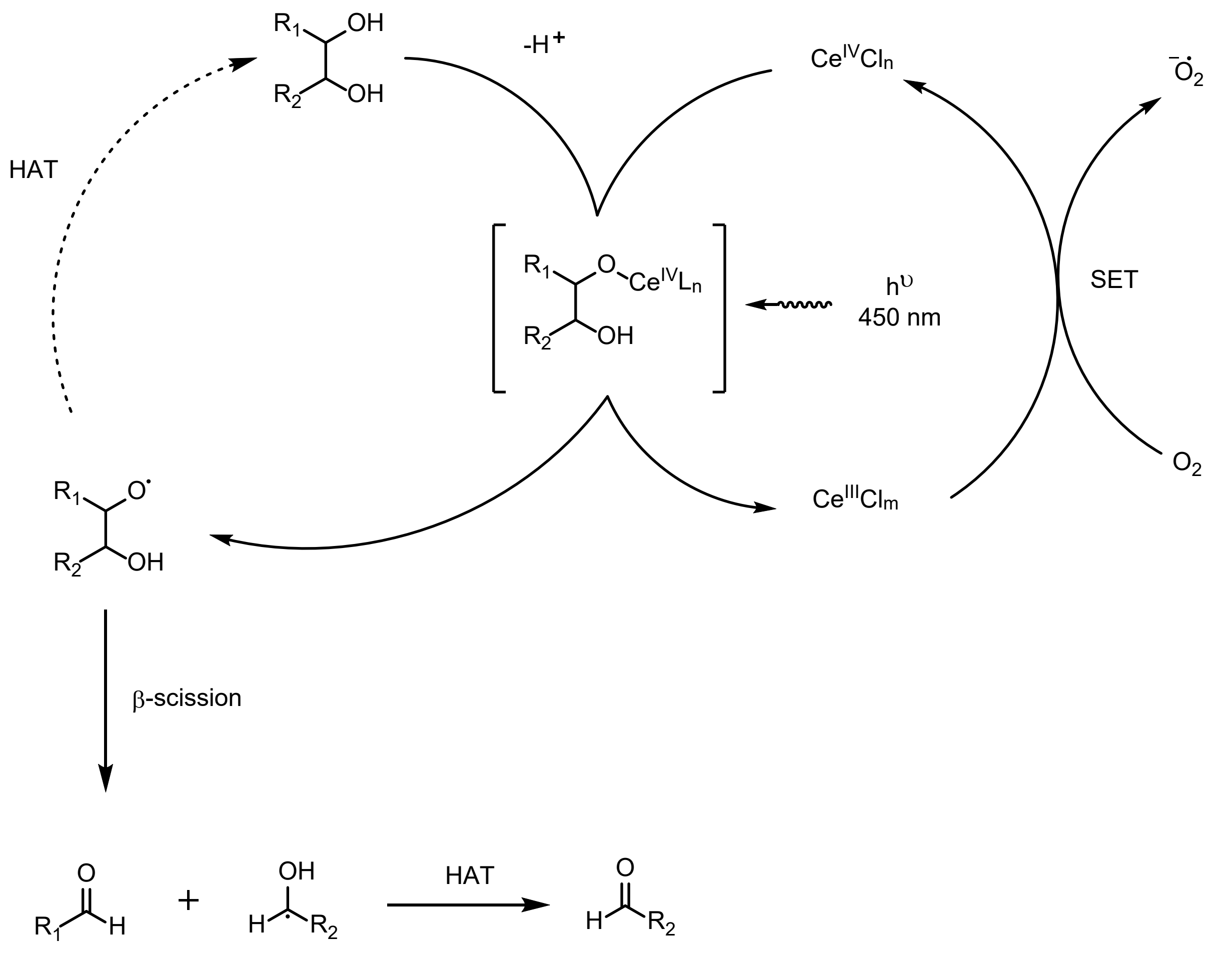

Figure S6. Proposed mechanism for the photocatalytic C–C bond cleavage of 1,2-diols (reproduced from literature[2]).

1.11 Kinetic study for depolymerization

A 1.0 mL vial was charged with 50 mg (0.16 mmol, 1.0 equiv. of repeating unit) of **P2**, 6.2 mg (0.016 mmol, 10 mol%) of catalyst, 23 mg (0.083 mmol, 50 mol%) of TBACl, MS, and 2.5 mL of HPLC-grade DMF (to follow the reaction progress by GPC). The vial was placed in a photoreactor equipped with a 450 nm LED and subjected to light irradiation for a predetermined time. The reaction progress was monitored at specific time intervals using gel permeation chromatography (GPC). The evolution of molecular weight and its distribution over time were summarized in the table below.

Table S2. Molecular weight and its distribution values over time during kinetic study

**Reaction time (min)**	**M_w (g/mol)[a]**	**M_n (g/mol)[a]**	**Đ[a]**
0	8,800	5,490	1.60
5	8,070	5,370	1.50
15	6,980	4,890	1.42
30	5,200	3,450	1.50
60	3,830	3,080	1.24
90	2,840	2,380	1.19
120	1,790	1,610	1.11

a)Determined by GPC in DMF calibrated with PMMA standard for crude samples.

1.12 Light *on*/*off* experiment

A 5.0 mL vial was charged with 80 mg of polymer **P5**, the corresponding amount of catalyst, dimethyl terephthalate as the internal standard, the reagent, activated MS, and deuterated dimethyl sulfoxide (DMSO-d_6) as the solvent. The LED light source was alternately switched *on* and *off* every 2 h for a total of 8 h. Following this period, the LED was switched *on* for 16 h, followed by a 2 h *off* period. NMR yields at each time point were determined by comparing the monomer and internal standard peaks in the ^{1}H-NMR spectra.

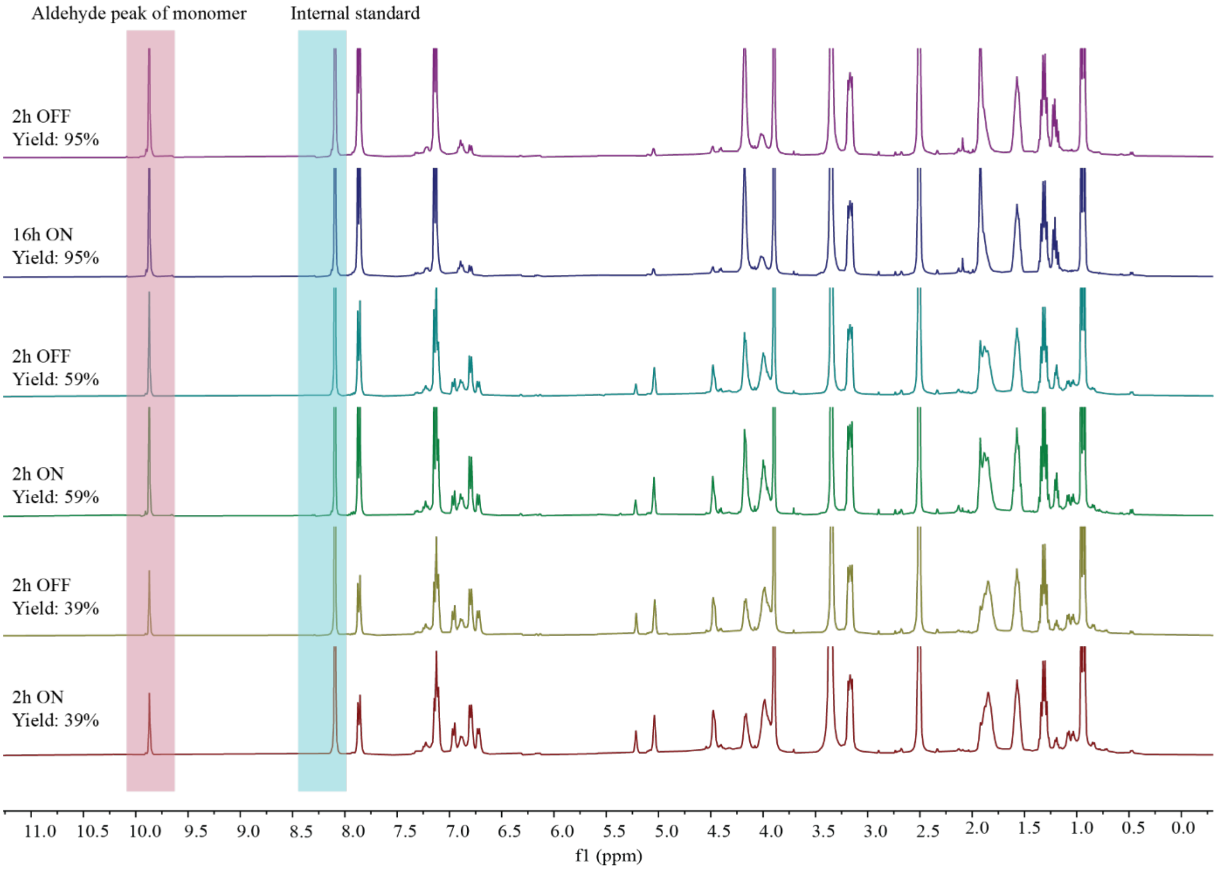

Figure S7. ^{1}H-NMR spectra of LED *on*/*off* experiments for **P5** in DMSO-d_6.

1.13 Closed-Loop Recycling

To proof the concept of closed-loop recycling, as-synthesized **P5** was used. 280 mg (0.93 mmol, 1.0 equiv. of repeating unit) of polymer, 127.84 mg (0.46 mmol, 0.5 equiv.) of TBACl, 34.5 mg (0.093 mmol, 0.01 equiv.) of $CeCl_3 \cdot 7H_2O$, molecular sieve (MS), and 14 mL of DMSO were added in a 20 mL vial. This solution was then split equally into 7x 4 mL vials, each equipped with a stirring bar, and located in the photoreactor. The solutions were illuminated with 450 nm LED light for 5.5 d. Reaction progress was followed by ^{1}H-NMR analysis based on polymer consumption as well as monomer generation. Upon completion of the reactions, the samples were combined and extracted 4x with $CHCl_3$/water to remove photocatalyst, TBACl, and DMSO. The chloroform phase was collected and the solvent was removed under reduced pressure. A quick filtration through a plug of silica gel using cyclohexane/EtOAc (1:1) as eluent was performed. After solvent removal and drying under reduced pressure, the recycled

monomer **M5** was obtained in 70% yield as a white solid and analyzed using ^{1}H-NMR spectroscopy (as shown in the manuscript).

Larger scale depolymerization: In a 20 mL vial, 300 mg of **P5** (0.99 mmol, 1.0 equiv. of repeating unit), 136.17 mg (0.49 mmol, 0.5 equiv.) of TBACl, 36.88 mg (0.099 mmol, 0.01 equiv.) of $CeCl_3 \cdot 7H_2O$, molecular sieve (MS), and 15 mL of DMSO were added. This vial was then located in the photoreactor equipped with a 450 nm LED. Light illumination was performed for 8 d. Same procedure was followed, as indicated above, for the work up. **M5** was obtained in 70% yield.

Closed-loop recycling: The recovered monomer was used to regenerate the corresponding polymer **P5**. 96 mg of monomer **M5** was dissolved in 4 mL of DMF and 2 equiv. of DIPEA were added, followed by degassing with argon. The mixture was located in the photoreactor, equipped with 365 nm LED and light irradiation was initiated for 18 h. Upon completion of reaction, excess DMF was removed under reduced pressure and the polymer was precipitated from MeCN. The precipitate was collected through vacuum filtration and dried overnight in a vacuum oven. The recycled polymer **P5** was characterized by ^{1}H-NMR spectroscopy, TGA, and DSC (as shown in the manuscript).

Orthogonal recycling: 15 mg of **P5** (0.050 mmol, 1.0 equiv. of repeating unit), 9.75 mg (0.050 mmol, 1.0 equiv.) of dimethyl terephthalate as an internal standard, 15 mg of polystyrene (PS), the corresponding amount of tetrabutylammonium chloride (TBACl), photocatalyst, molecular sieve, and 0.75 mL of DMSO-d_6 were added in a 1.0 mL vial equipped with a stirring bar. Locating the vial in the photoreactor equipped with 450 nm LED was followed by light irradiation. Upon completion of the reaction, PS pieces were removed and washed with acetone and dried over vacuum oven overnight. Furthermore, sample were taken from reaction mixture to calculate the monomer yield via NMR analysis in DMSO-d_6. The NMR yield was calculated by comparing the aromatic peak of the internal standard and the aldehyde peak of the product **M5**. Yield of **M5** was calculated as 81% (Figure S8).

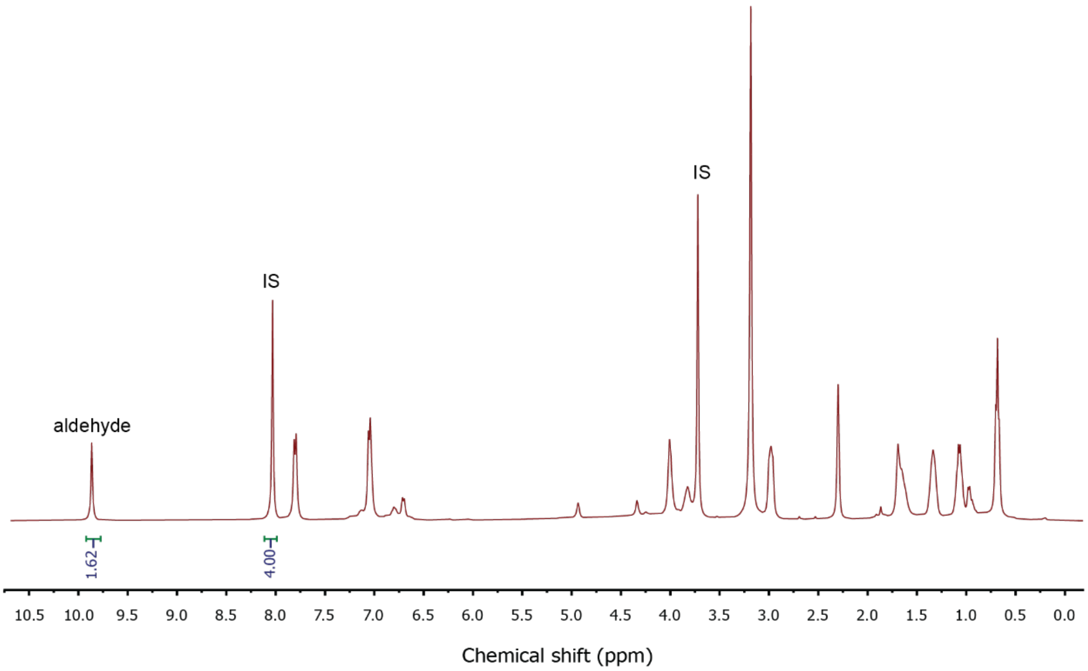

Figure S8. ^{1}H-NMR spectrum of orthogonal recycling approach in the presence of PS additive, dimethyl terephthalate as internal standard (DMSO-d_6, 400 MHz).

References

[1] F. Yang, F. Xie, Y. Zhang, Y. Xia, W. Liu, F. Jiang, C. Lam, Y. Qiao, D. Xie, J. Li, L. Fu, *Bioorg. Med. Chem. Let.* **2017**, *27*, 2166.

[2] J. Schwarz, B. König, *Chem. Commun.* **2019**, *55*, 486.

Appendix

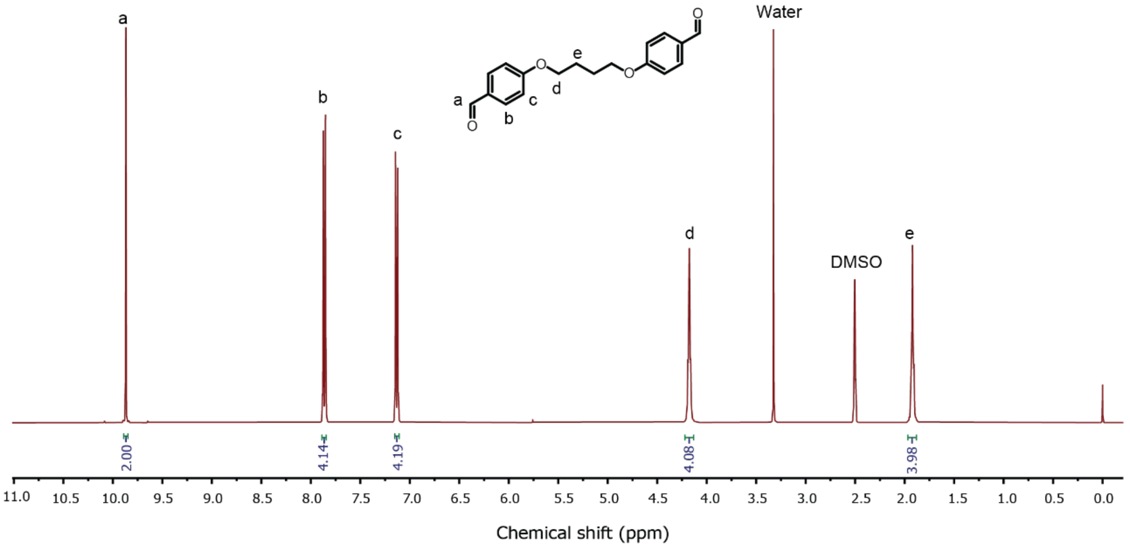

Figure S9. ^{1}H-NMR spectrum of **M5** (DMSO-d_6, 400 MHz).

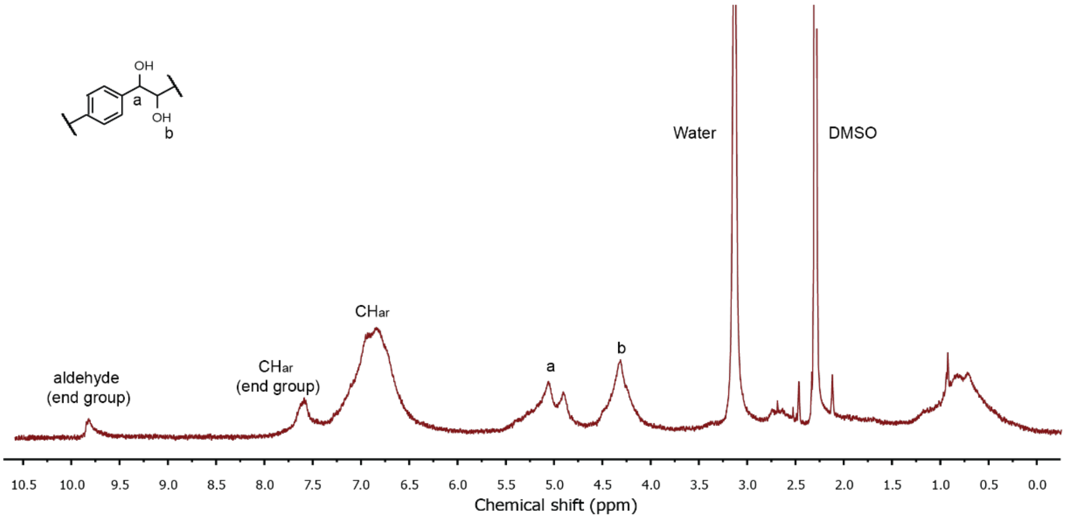

Figure S10. ^{1}H-NMR spectrum of **P1** (DMSO-d_6, 400 MHz).

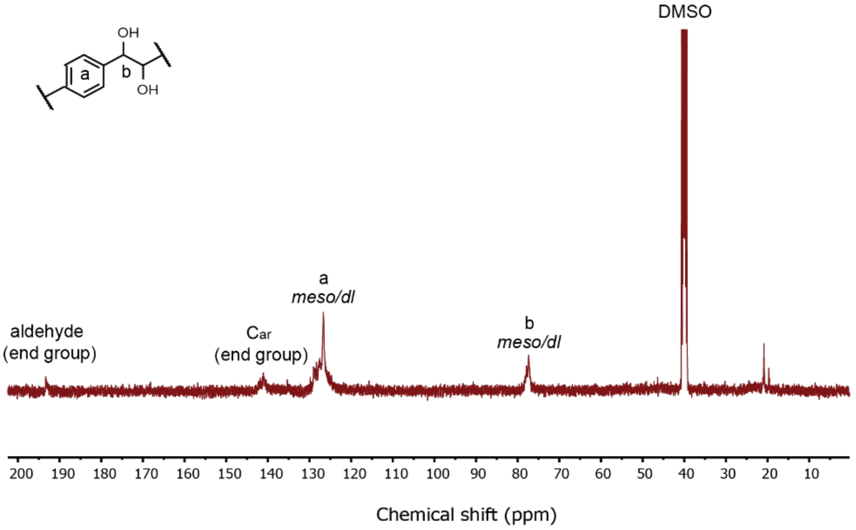

Figure S11. ^{13}C-NMR spectrum of **P1** (DMSO-d_6, 100 MHz).

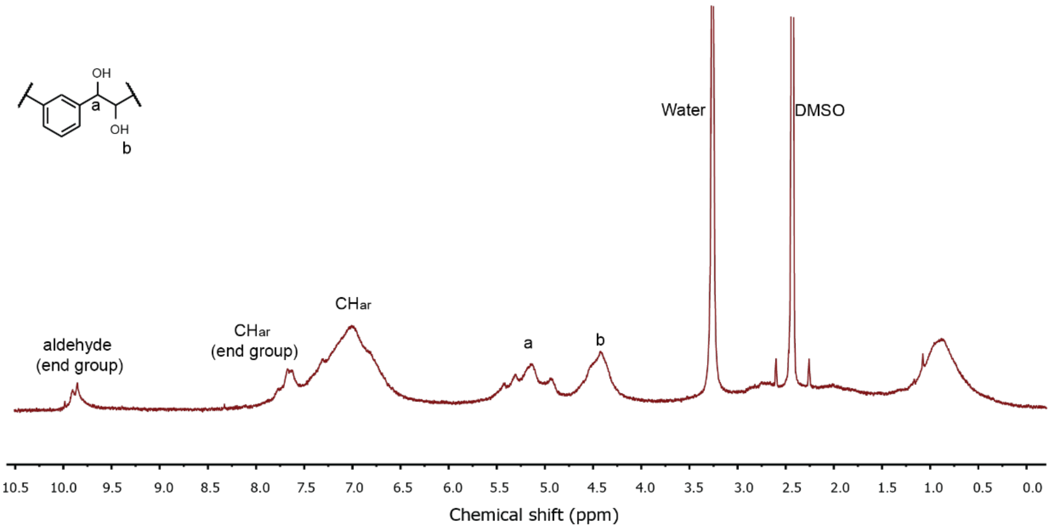

Figure S12. ^{1}H-NMR spectrum of **P2** (DMSO-d_6, 400 MHz).

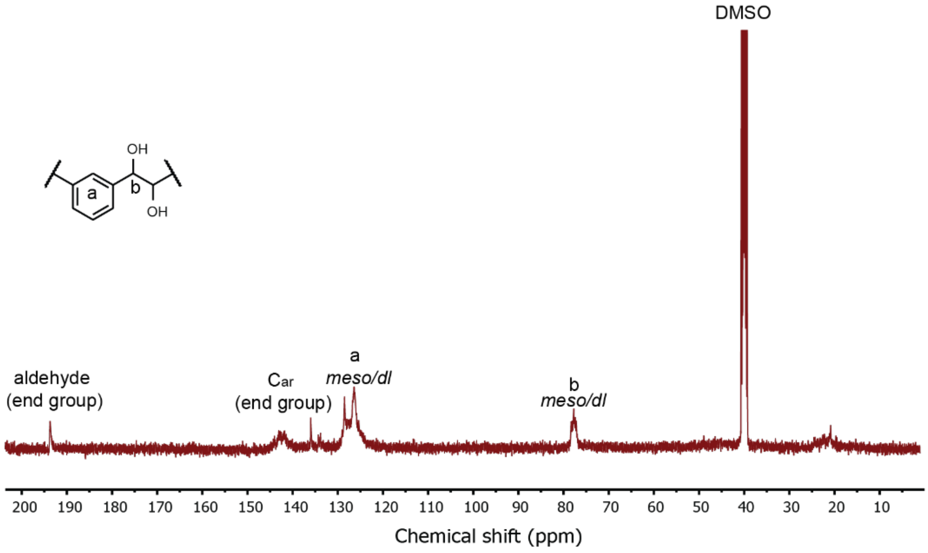

Figure S13. ^{13}C-NMR spectrum of **P2** (DMSO-d_6, 100 MHz).

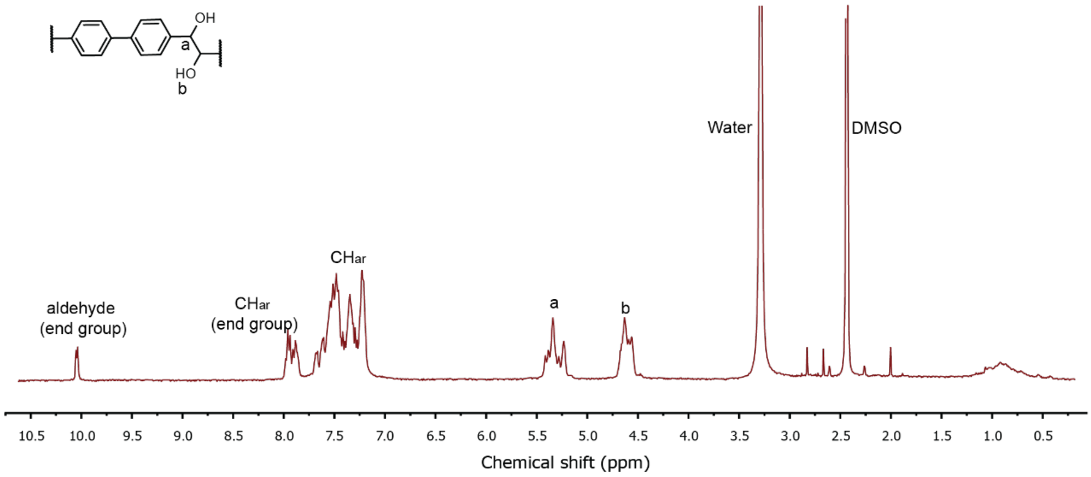

Figure S14. ^{1}H-NMR spectrum of **P3** (DMSO-d_6, 400 MHz).

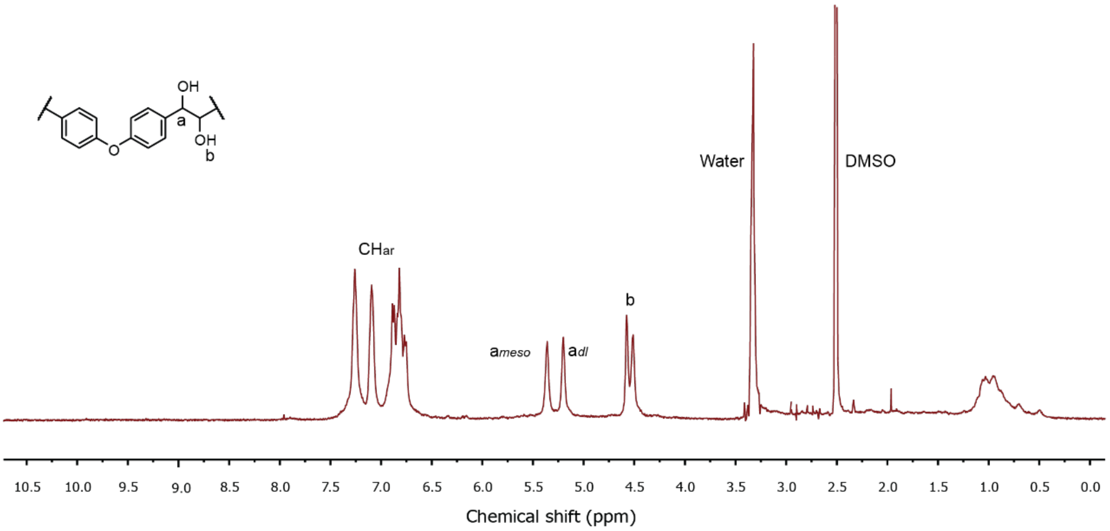

Figure S15. ^{1}H-NMR spectrum of **P4** (DMSO-d_6, 400 MHz).

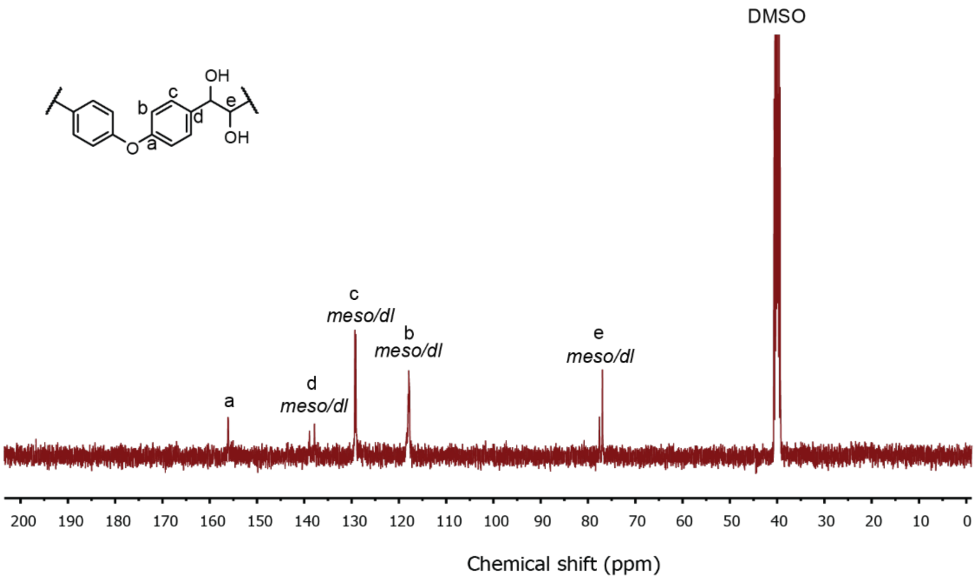

Figure S16. ^{13}C-NMR spectrum of **P4** (DMSO-d_6, 100 MHz).

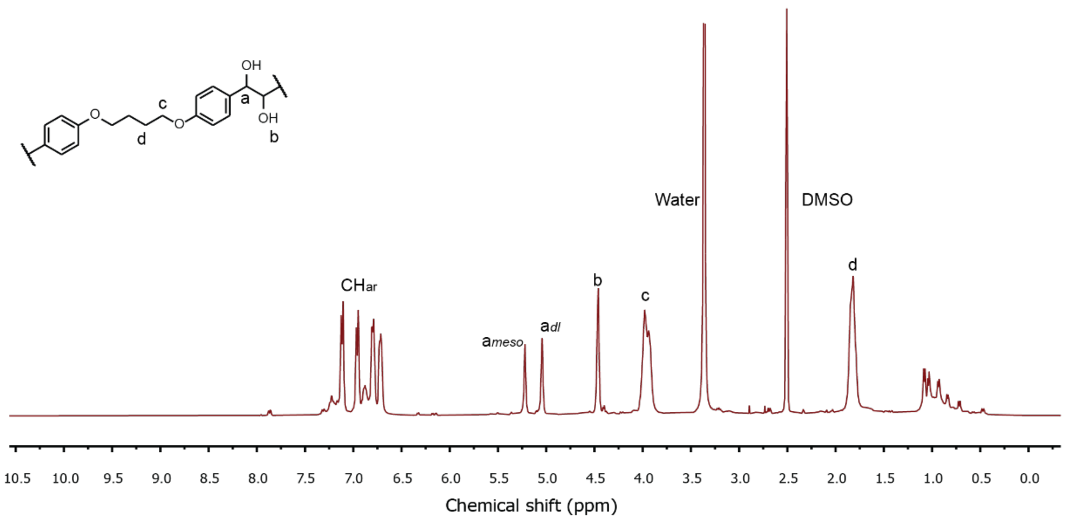

Figure S17. ^{1}H-NMR spectrum of **P5** (DMSO-d_6, 400 MHz).

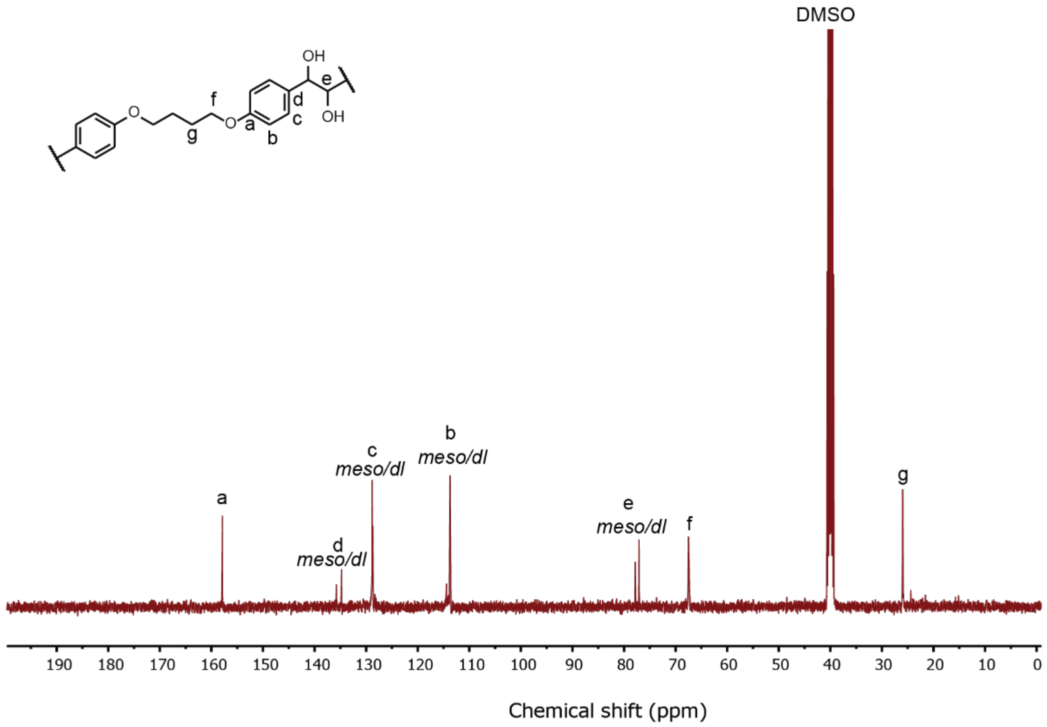

Figure S18. ^{13}C-NMR spectrum of **P5** (DMSO-d_6, 100 MHz).

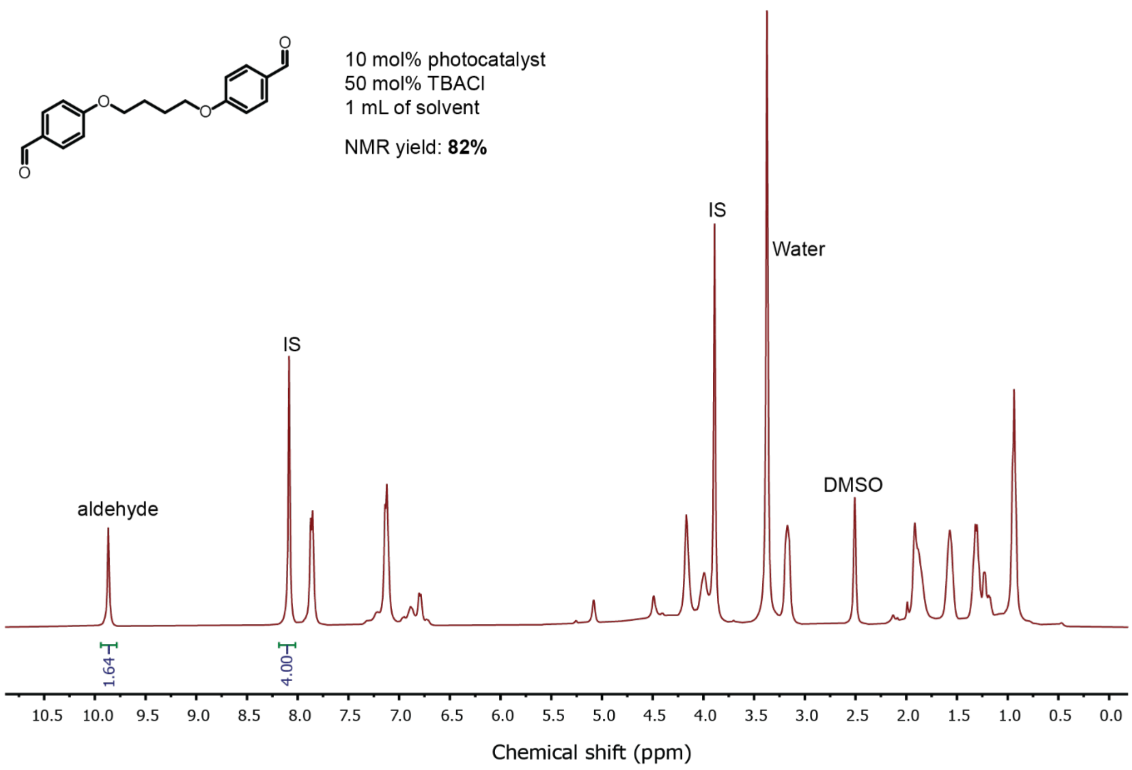

Figure S19. ^{1}H-NMR spectrum of Entry 1 (Table 2 in manuscript) for depolymerization of **P5**, dimethyl terephthalate as internal standard (IS) (DMSO-d_6, 400 MHz).

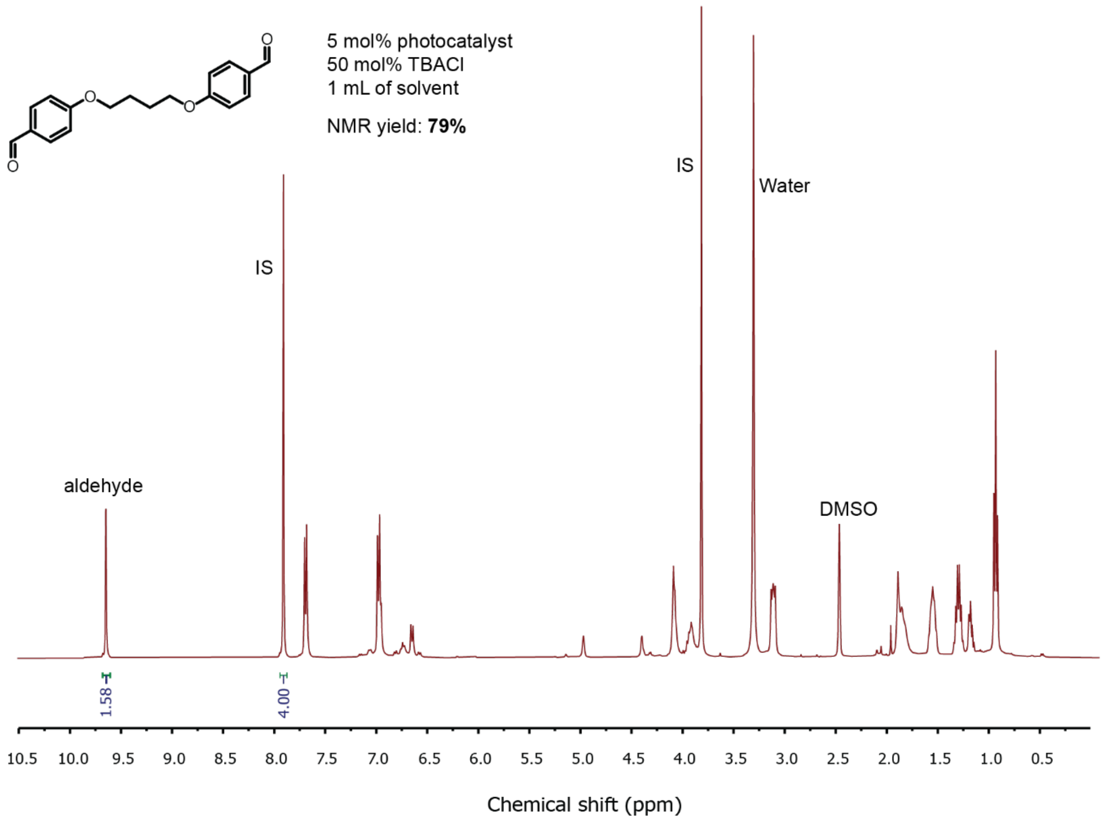

Figure S20. ^{1}H-NMR spectrum of Entry 2 (Table 2 in manuscript) for depolymerization of **P5**, dimethyl terephthalate as internal standard (IS) (DMSO-d_6, 400 MHz).

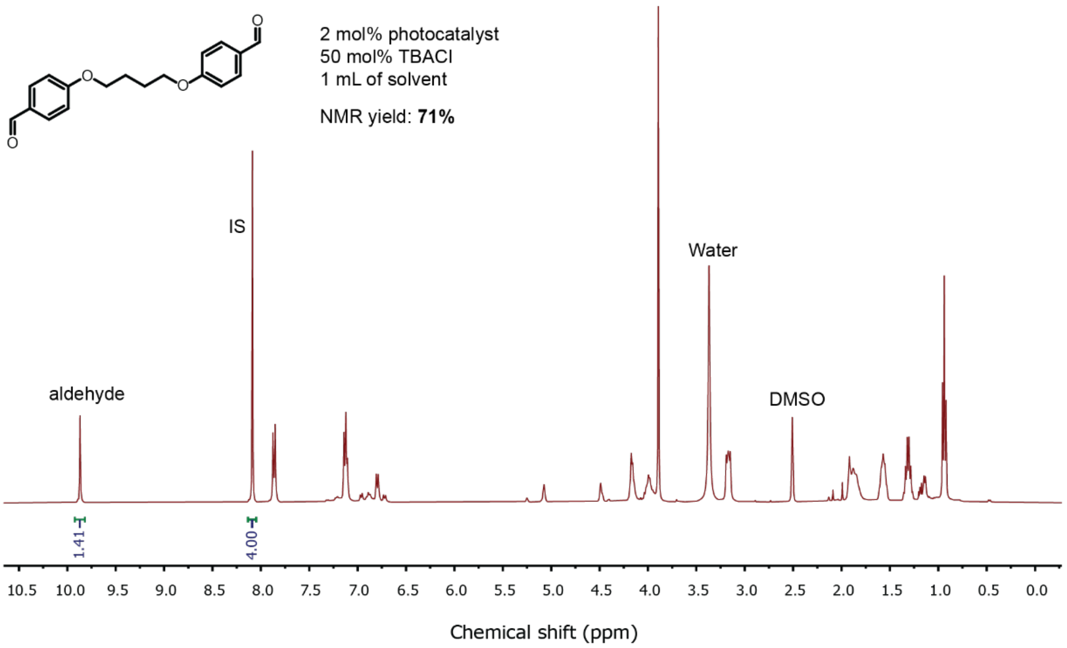

Figure S21. ^{1}H-NMR spectrum of Entry 3 (Table 2 in manuscript) for depolymerization of **P5,** dimethyl terephthalate as internal standard (IS) (DMSO-d_6, 400 MHz).

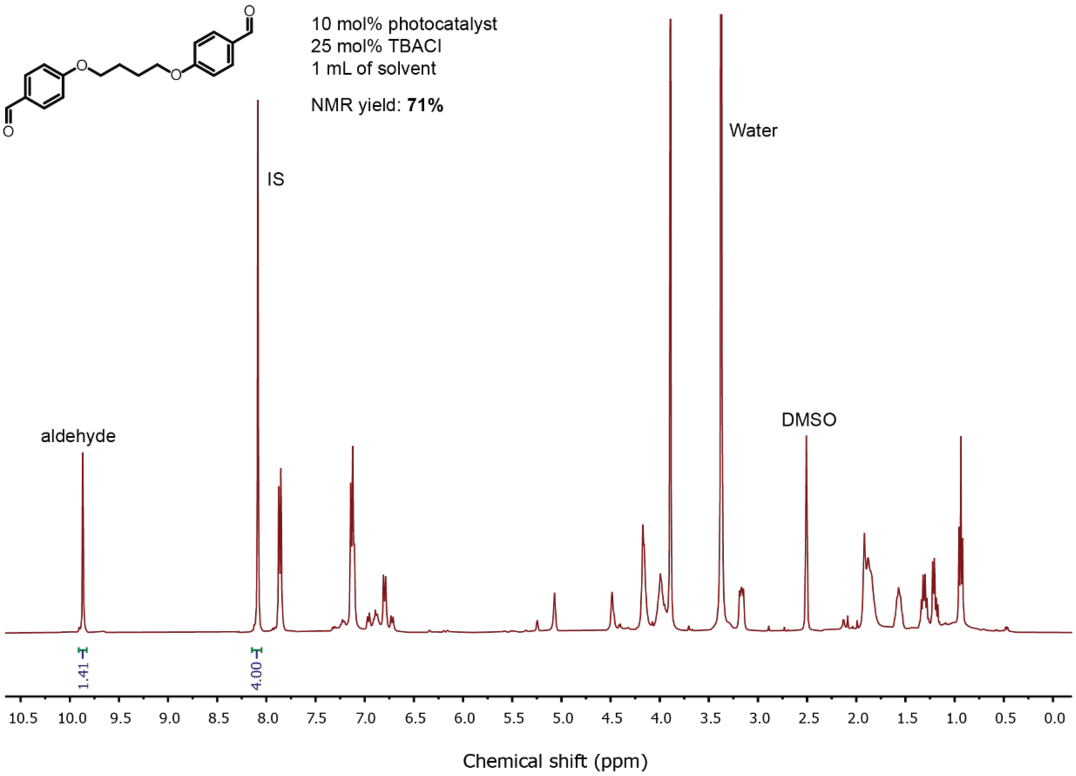

Figure S22. ^{1}H-NMR spectrum of Entry 4 (Table 2 in manuscript) for depolymerization of **P5**, dimethyl terephthalate as internal standard (IS) (DMSO-d_6, 400 MHz).

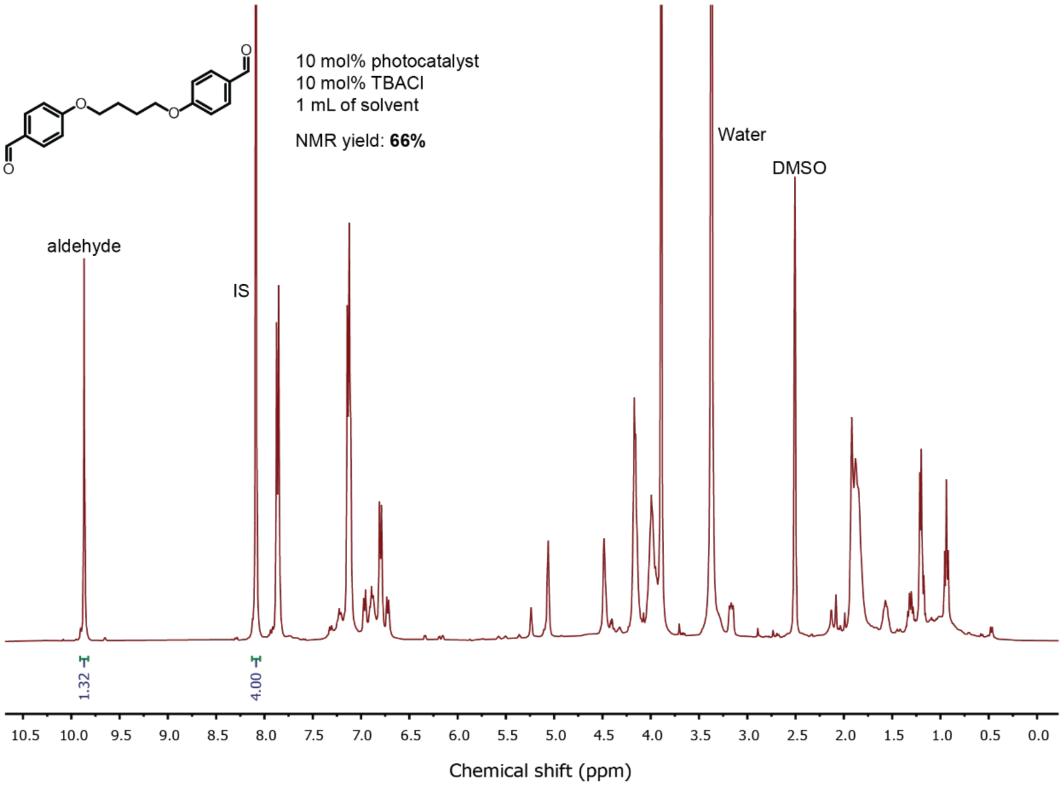

Figure S23. ^{1}H-NMR spectrum of Entry 5 (Table 2 in manuscript) for depolymerization of **P5**, dimethyl terephthalate as internal standard (IS) (DMSO-d_6, 400 MHz).

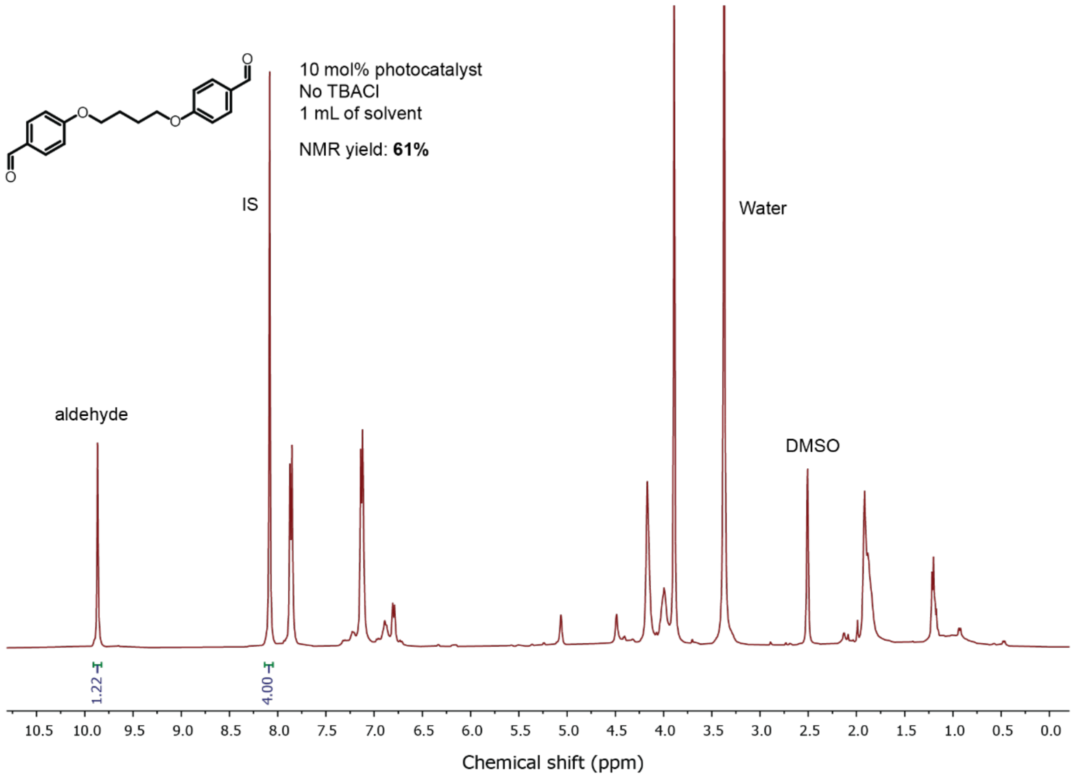

Figure S24. ^{1}H-NMR spectrum of Entry 6 (Table 2 in manuscript) for depolymerization of **P5**, dimethyl terephthalate as internal standard (IS) (DMSO-d_6, 400 MHz).

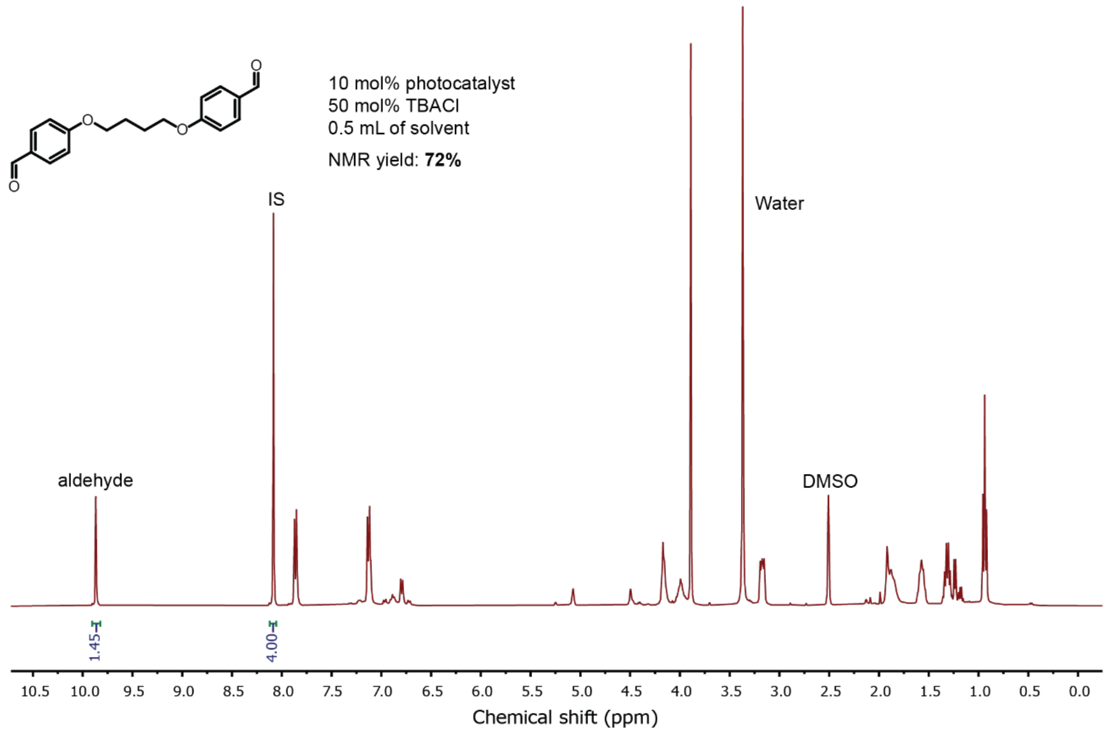

Figure S25. ^{1}H-NMR spectrum of Entry 7 (Table 2 in manuscript) for depolymerization of **P5**, dimethyl terephthalate as internal standard (IS) (DMSO-d_6, 400 MHz).

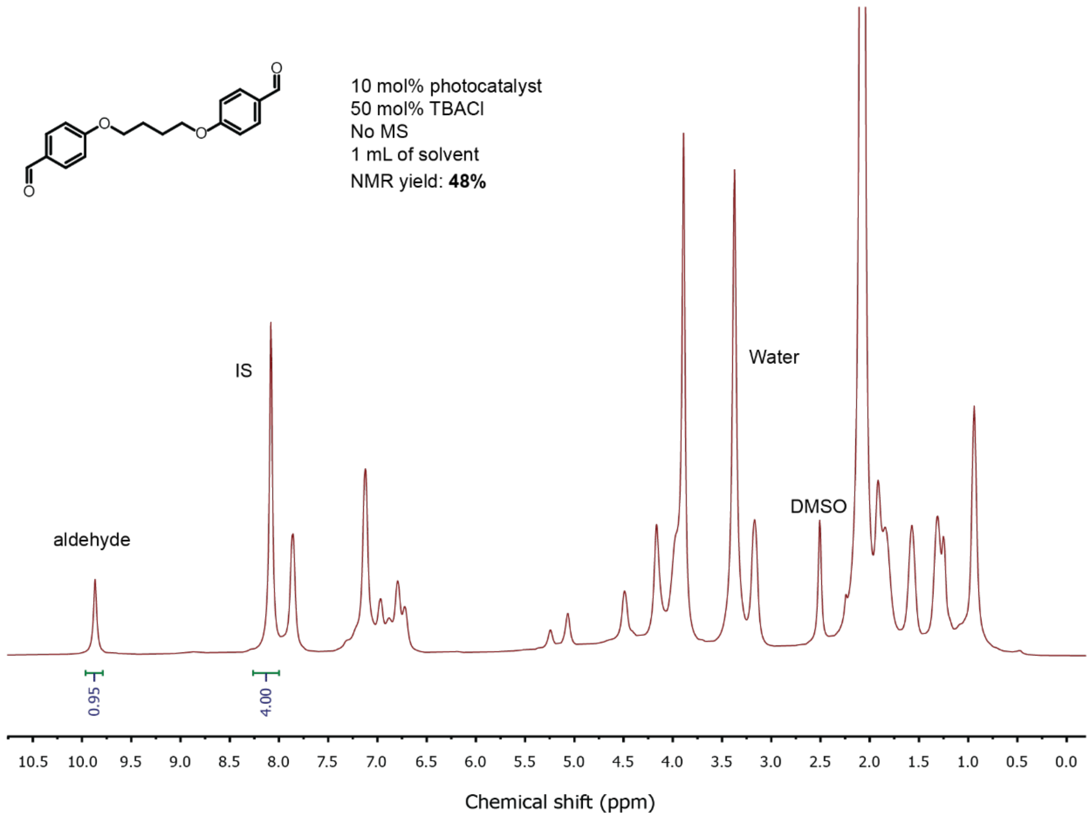

Figure S26. ^{1}H-NMR spectrum of Entry 8 (Table 2 in manuscript) for depolymerization of **P5**, dimethyl terephthalate as internal standard (IS) (DMSO-d_6, 400 MHz).

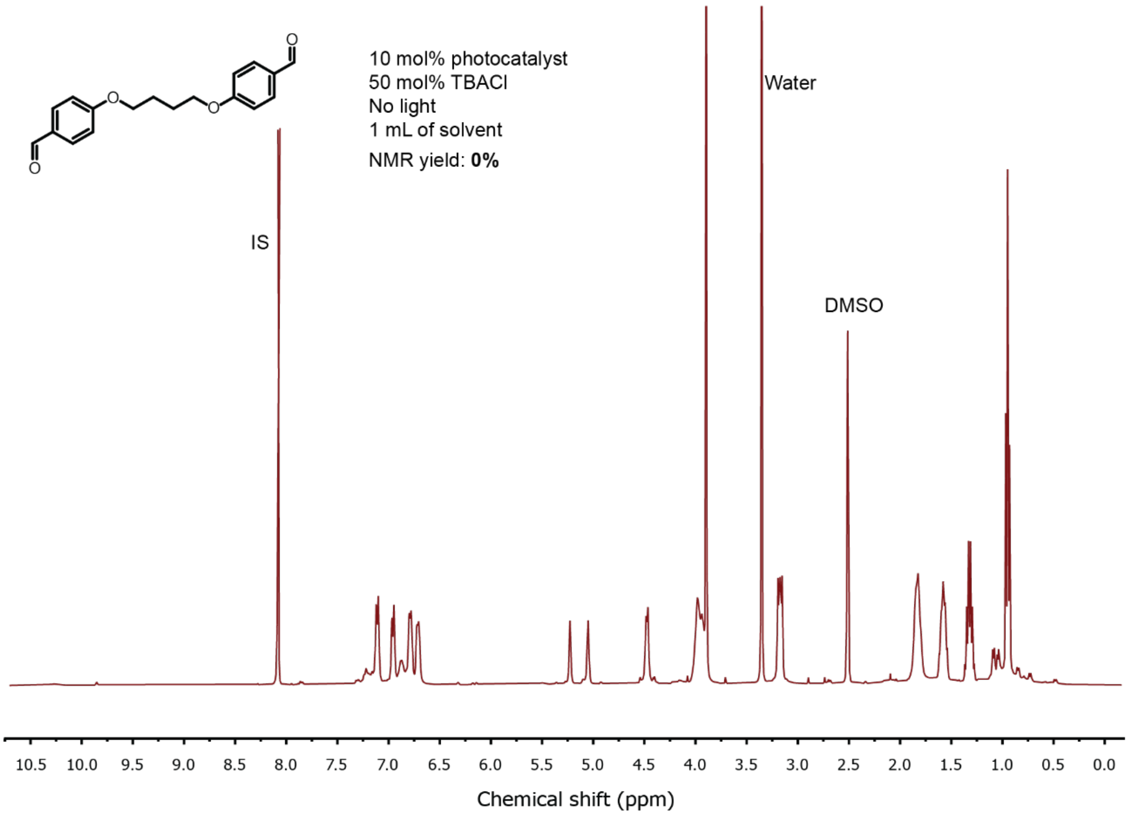

Figure S27. ^{1}H-NMR spectrum of Entry 9 (Table 2 in manuscript) for depolymerization of **P5**, dimethyl terephthalate as internal standard (IS) (DMSO-d_6, 400 MHz).

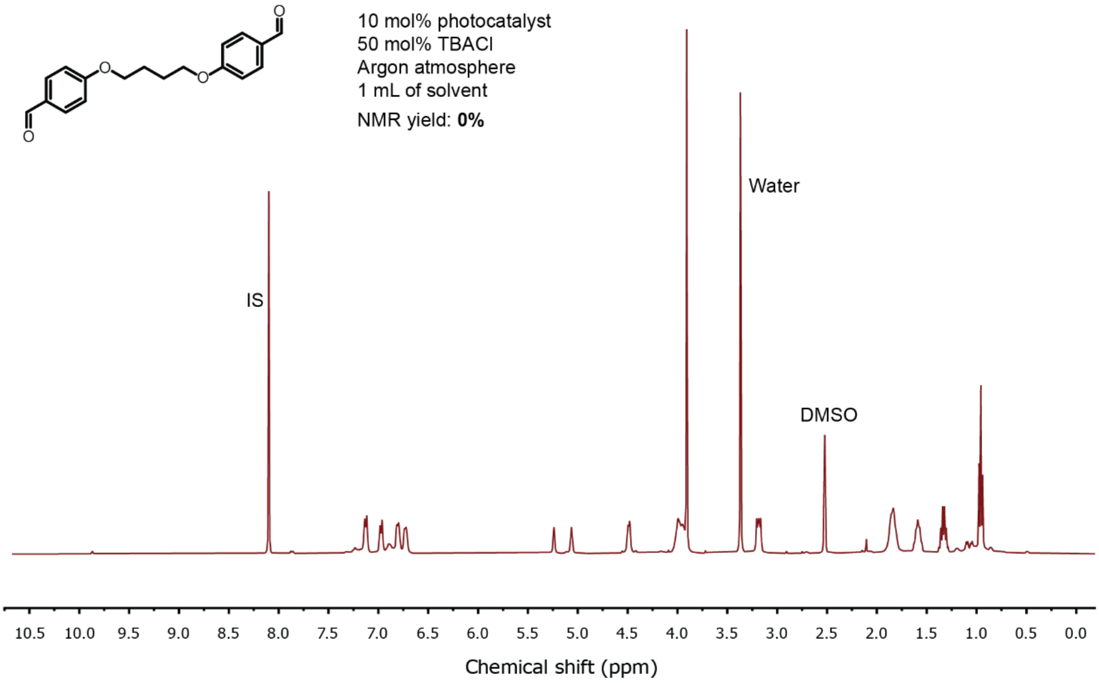

Figure S28. ^{1}H-NMR spectrum of Entry 10 (Table 2 in manuscript) for depolymerization of **P5**, dimethyl terephthalate as internal standard (IS) (DMSO-d_6, 400 MHz).
